# Exploring Bismuth Coordination Complexes as Visible-Light Absorbers: Synthesis, Characterization, and Photophysical Properties

**DOI:** 10.1021/acs.inorgchem.3c03290

**Published:** 2023-12-15

**Authors:** Harsh Bhatia, Junjun Guo, Christopher N. Savory, Martyn Rush, David Ian James, Avishek Dey, Charles Chen, Dejan-Krešimir Bučar, Tracey M. Clarke, David O. Scanlon, Robert G. Palgrave, Bob C. Schroeder

**Affiliations:** † Department of Chemistry, University College London, 20 Gordon Street, London WC1H 0AJ, United Kingdom; ‡ Thomas Young Centre, University College London, London WC1E 6BT, United Kingdom; § Polysolar Ltd, High Cross, Aurora Cambridge at BAS, Madingley Rd, Cambridge CB3 0ET, United Kingdom; ∥ Johnson Matthey Technology Centre, Blount’s Court, Sonning Common, Reading RG4 9NH, United Kingdom; ⊥ Diamond Light Source Ltd., Diamond House, Harwell Science and Innovation Campus, Didcot, Oxfordshire OX11 0DE, United Kingdom

## Abstract

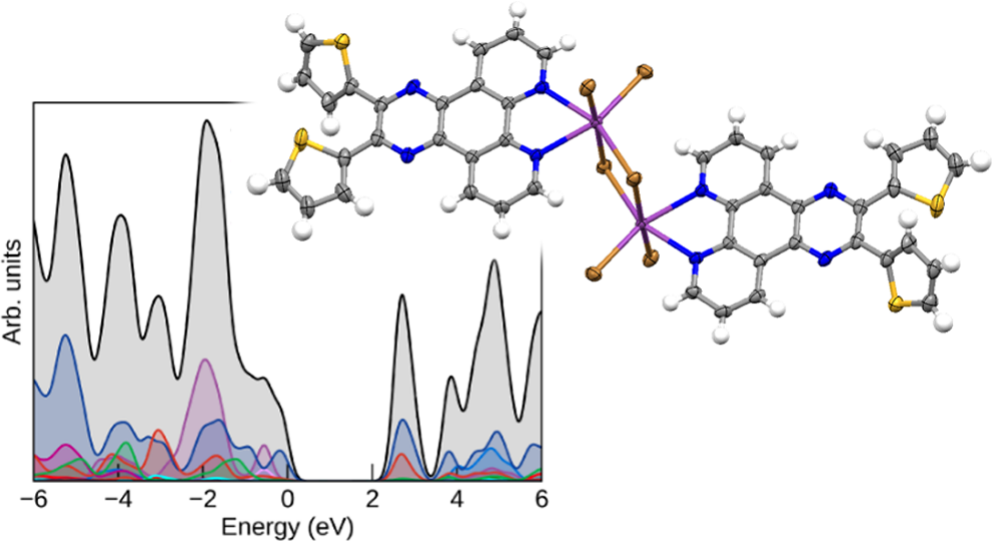

Bismuth-based coordination complexes are advantageous over other metal complexes, as bismuth is the heaviest nontoxic element with high spin–orbit coupling and potential optoelectronics applications. Herein, four bismuth halide-based coordination complexes [Bi_2_Cl_6_(phen-thio)_2_] (**1**), [Bi_2_Br_6_(phen-thio)_2_] (**2**), [Bi_2_I_6_(phen-thio)_2_] (**3**), and [Bi_2_I_6_(phen-Me)_2_] (**4**) were synthesized, characterized, and subjected to detailed photophysical studies. The complexes were characterized by single-crystal X-ray diffraction, powder X-ray diffraction, and NMR studies. Spectroscopic analyses of **1**–**4** in solutions of different polarities were performed to understand the role of the organic and inorganic components in determining the ground- and excited-state properties of the complexes. The photophysical properties of the complexes were characterized by ground-state absorption, steady-state photoluminescence, microsecond time-resolved photoluminescence, and absorption spectroscopy. Periodic density functional theory (DFT) calculations were performed on the solid-state structures to understand the role of the organic and inorganic parts of the complexes. The studies showed that changing the ancillary ligand from chlorine (Cl) and bromine (Br) to iodine (I) bathochromically shifts the absorption band along with enhancing the absorption coefficient. Also, changing the halides (Cl, Br to I) affects the photoluminescent quantum yields of the ligand-centered (LC) emissive state without markedly affecting the lifetimes. The combined results confirmed that ground-state properties are strongly influenced by the inorganic part, and the lower-energy excited state is LC. This study paves the way to design novel bismuth coordination complexes for optoelectronic applications by rigorously choosing the ligands and bismuth salt.

## Abstract 2

We synthesized four new Bismuth halide-based coordination complexes revealing that altering the ancillary ligand not only shifts the absorption band, but also impacts photoluminescent quantum yields of the ligand-centered emissive state without significantly affecting lifetimes. This confirms the strong influence of the inorganic component on ground state properties, with the lower energy excited state being ligand-centered. This suggests opportunities for designing novel Bismuth coordination complexes for optoelectronic applications by carefully selecting ligands and Bismuth salts.

## Introduction

Coordination complexes of main group metals with ns^2^ lone-pair electrons offer a variety of stereochemical activities, due to which the main group metals show a rich coordination chemistry.^1−3^ The possibility of multiple bonds and deformation of the coordination sphere lead the complexes to show a diverse structure–property relationship for functional materials.^4^ This deformation in the structure may lead to multiple potential applications, which include varied optical properties and catalysis.^5−8^ Until now, the most reported and well-explored metal complexes are the 3d and 4d series transition metals, and their chemistry and photophysics are well understood.^9,10^ Common perception implies that transition metals possess a richer chemistry than the main group metals, due to which the properties of these main group metals are often overlooked. In comparison to transition metals, main group metal coordination complexes are underexplored as they are usually diamagnetic, and the valence orbitals involved are s or p.^1^ The possibility for the formation of multiple coordination environments, limited solubility, and dissociation of the complexes in solution has hindered their studies.

The main group metal bismuth exists in different oxidation states (Bi^+^, Bi^2+^, Bi^3+^, Bi^5+^), with Bi^3+^ being the most stable ionic form, often observed in hybrid organic–inorganic compounds, doped inorganic lattices, and perovskites.^11−13^ The luminescent properties of Bi^3+^ were extensively studied due to its broad luminescence feature in the visible and near-IR regions,^11,12,14^ while detailed spectroscopic studies are absent for bismuth-based coordination complexes. Recently, bismuth (Bi)-based coordination complexes in an oxidation state of +3 have attracted significant interest from the scientific community to explore their optoelectronic properties, as bismuth has a high spin–orbit coupling (SOC) constant, is nontoxic among all its neighbors, and inexpensive.^15−17^ Additionally, the inherently richer structural diversity due to the stereochemically active 6s^2^ lone pair of electrons can be controlled to harness multiple complexes with varied coordination numbers.^18−27^ The influence of lone pairs on the physicochemical properties makes Bi an intriguing material candidate for numerous applications like organic light-emitting diodes (OLEDs), photocatalysis, and solar cells. Such structural diversity led bismuth to possess multiple structures with varied alignments of the electronic ground- and excited-state properties. Several bismuth-containing small coordination complexes and coordination polymers were reported; however, research was primarily limited to synthesis, structural description, and the supramolecular interactions in the complexes.^28−30^ The detailed mechanism of photophysical behavior considering the structure–property relationship and the role of main ligand(s), the ancillary ligand (chlorine, bromine, and iodine), and bismuth metal is still not well understood and hence requires a systematic study to develop hybrid organic–inorganic systems able to challenge more widely used transition-metal complexes.

**Figure 1:**
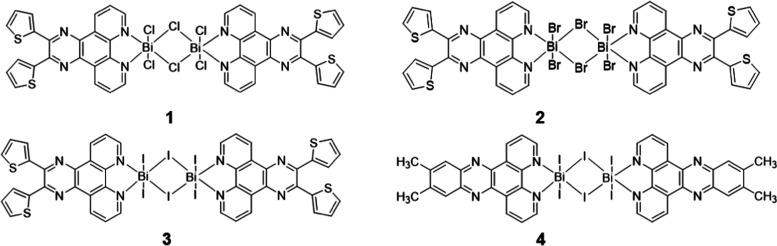
Molecular structures of coordination complexes of **1**, **2**, **3**, and **4**.

Therefore, in this work, we report the comparative study of bismuth coordination complexes, [Bi_2_Cl_6_(phen-thio)_2_] (**1**), [Bi_2_Br_6_(phen-thio)_2_] (**2**), [Bi_2_I_6_(phen-thio)_2_] (**3**), and [Bi_2_Cl_6_(phen-Me)_2_] (**4**) (Figure 1). Detailed photophysical studies reveal the distinct contributions of the organic and inorganic parts in controlling and tuning the photophysical properties of the ground and excited states of the complexes. The experiments show that the ground-state properties are strongly controlled by the inorganic part of the complexes, while the emissive excited state is ligand-based. Additionally, the studies show that the choice of the ancillary ligand significantly affects the excited-state property of the emissive state. The presence of BiI_3_ as the inorganic core leads to the enhancement in the ground-state absorption along with the deactivation of the emissive excited state due to the presence of a lower-energy Bi–I-based ligand-to-metal charge transfer (LMCT) excited state. Through the initial studies, we hypothesize that BiI_3_-based coordination complexes could prove to be highly beneficial for applications involving the absorption of visible light.

## Experimental Section

### General Information

BiCl_3_, BiBr_3_, BiI_3_, and hydrazine hydrate were obtained from Sigma-Aldrich. DMF-*d*_7_ was purchased from Sigma-Aldrich. All other reagents were purchased from Fluorochem and Apollo Scientific and used without further purification. HPLC-grade dimethylformamide (DMF), acetonitrile (ACN), and tetrahydrofuran (THF) were purchased from Sigma-Aldrich for photophysical analysis.

### Spectroscopic Measurements

^1^H NMR and ^13^C NMR spectroscopy was performed with either an AVANCE III 600 or an Avance Neo 500 instrument at room temperature. The chemical shifts δ are given in parts per million (ppm) and referenced to DMSO-*d*_6_ (2.50 ppm in ^1^H NMR and 39.52 ppm in ^13^C NMR spectroscopy), CDCl_3_ (7.26 ppm in ^1^H NMR and 77.16 ppm in ^13^C NMR spectroscopy), and DMF-*d*_7_ (8.03, 2.92, 2.75 ppm in ^1^H NMR). All of the samples for photophysical studies were prepared as stock solutions in DMF and further diluted in ACN and THF for the measurements. The ground-state absorption spectra for all samples were collected by using a Shimadzu UV-3600i Plus spectrophotometer under ambient conditions. The solution UV–vis spectra were recorded in quartz cells with a path length of 10 mm. The photoluminescence emission and excitation spectra were measured on a Fluorolog-3 spectrometer (Horiba) with a 0.2 s integration time and reference corrected for an excitation light source and emission spectral response. The emission lifetimes of the samples were measured on a Lifespec II (Edinburgh Instruments) time-correlated single photon counting (TCSPC) fluorescence lifetime spectrometer using a 375.4 nm pulsed laser diode. A pump–probe micromillisecond transient absorption (TA) spectroscopy setup was used to measure the TA spectra and kinetics. Laser pulses (repetition rate 10 Hz, pulse duration 6 ns) were generated by a Nd:YAG laser (Spectra Physics, INDI-40-10). Excitation wavelengths were selected by a versaScan L-532 OPO, and the excitation density was set in the range between 0.3 and 120 mJ cm^–2^ using neutral density filters, measured by an ES111C power meter (Thorlabs). The probe light was provided by a quartz tungsten halogen lamp (IL1, Bentham). Probe wavelength selectivity was achieved by using bandpass filters and a Cornerstone 130 monochromator (Oriel Instrument) before the detector. The TA signals were recorded with Si and InGaAs photodiodes. The signal from the photodiodes was preamplified and sent to the main amplification system via an electronic filter (Costronic Electronics), which was connected to an oscilloscope (Tektronics, DPO4034 B) and PC. The degassed solutions for TA spectroscopy were prepared using three repeat freeze–pump–thaw cycles. The concentration of the samples was chosen to maintain the optical density of 0.2 contained within the 0.2 cm path-length quartz cuvette.

### X-ray Diffraction

The diffraction data for **1**–**4** were collected on a four-circle Agilent SuperNova (Dual Source) single-crystal X-ray diffractometer using a microfocus Cu Kα X-ray beam (λ = 1.54184 Å) and an Atlas CCD plate detector (in case of **1** and **2**) or a HyPix-Arc 100 hybrid pixel array detector (in case of **3** and **4**). The sample temperatures were controlled with an Oxford Instruments cryojet. All data were processed using the CrysAlis^Pro^ program package from Rigaku Oxford Diffraction. The crystal structures were solved with the SHELXT program,^31^ used within the Olex2 software suite,^32^ and refined by least-squares on the basis of *F*^2^ with the SHELXL^33^ program using the ShelXle graphical user interface.^34^ All nonhydrogen atoms were refined anisotropically by the full-matrix least-squares method. Hydrogen atoms associated with carbon atoms were refined isotropically [*U*_iso_(H) = 1.2*U*_eq_(C)] in geometrically constrained positions. The *F*_0_–*F*_c_ difference maps were used in all cases to identify disordered thiophene moieties. These disorders were modeled using the SAME similarity restraint command in SHELXL.^33^ The anisotropic parameters of the disordered thiophene groups were restrained or constrained using the SIMU or EADP commands in SHELXL.^33^ The crystallographic and refinement parameters of **1**–**4** are given in Table S1.

### Computational Methods

Periodic density functional theory (DFT) calculations were performed on the solid-state structures of the three hybrid inorganic–organic complexes within the Vienna Ab initio Simulation Package (VASP).^35−38^ The projector augmented wave method was used to describe the interactions between core and valence electrons, using scalar-relativistic pseudopotentials for all atoms.^39^ The Bi 5d electrons were included in the valence set. Due to the size and complexity of the crystal structures of the three compounds, GGA DFT was used for geometric relaxation—the Perdew, Burke, and Ernzerhof (PBE) functional^40^ with the addition of Grimme’s D3 (PBE+D3) correction^41^ as well as the revised PBE functional for solids (PBEsol)^42^ were used for this purpose due to their prior success in describing the crystal structures of extended organic–inorganic complexes.^43−47^ As semiconductor band gaps are severely underestimated in GGA DFT, the hybrid DFT functional HSE06 was used for the calculation of the electronic structures of the three systems.^48,49^ Spin–orbit coupling was additionally included to evaluate the relativistic renormalization of the band structure in each case (HSE06+SOC).

For each structure, the *k*-point mesh and cutoff energy for the plane wave basis set were converged using a criterion of 1 meV per atom for the total energy. A cutoff energy of 500 eV was found to be sufficient for all three systems, and *k*-meshes of 2 × 2 × 3, 3 × 2 × 2, and 3 × 1 × 1 were found to be sufficient for the standardized primitives of the iodide, bromide, and chloride compounds, respectively. All structures were optimized using GGA DFT initially until the forces on each atom did not exceed 0.01 eV/Å.

Ultraviolet photoelectron spectroscopy (UPS) measurements were recorded on a Thermo Scientific Theta-Probe spectrometer with a helium discharge lamp emitting in the ultraviolet range (HeI = 21.22 eV), and the samples were biased at −9 V.

### Synthesis

#### Ligand Synthesis

##### 1,10-Phenanthroline-5,6-dioxime

The reaction was carried out following the slightly modified literature procedure.^50,51^ 1,10-Phenanthroline-5,6-dione (3 g, 14.27 mmol) and Na_2_CO_3_ (2.3 g, 21.41 mmol) were added to ethanol (210 mL) and heated to reflux for 30 min. Alongside, hydroxylamine hydrochloride (3.47 g, 49.95 mmol) was dissolved in ethanol (75 mL) by heating the solution to 50 °C and added dropwise to the hot solution of 1,10-phenanthroline-5,6-dione. The reaction mixture was refluxed for 5 h, and on cooling, a yellow precipitate formed, which was decanted to another flask without collecting the black solid. The solvent was removed under a vacuum, and the resulting solid was washed with water and THF. The yellow precipitate obtained was kept in a vacuum oven to dry at 40 °C overnight to give 2.62 g (10.89 mmol) of the product with a yield of 76%. The product obtained was used without further purification for the next reaction. HRMS (ESI) *m*/*z*: 241.0776, calculated for [M + H^+^] = 241.0721.

##### 5,6-Diamino-1,10-phenanthroline

The reduction of 1,10-phenanthroline-5,6-dioxime was carried out following the slightly modified literature procedure.^50,51^ A mixture of 1,10-phenanthroline-5,6-dioxime (1.20 g, 5 mmol), Pd/C (0.13 g), and 180 mL of ethanol was purged with nitrogen for 30 min and subsequently refluxed under a nitrogen atmosphere for another 30 min. Alongside, hydrazine hydrate (N_2_H_4_·H_2_O (50–60%)) (12.1 mL, 149.86 mmol) was also purged with nitrogen for 30 min and added dropwise for over a period of 1 h to a hot solution of 1,10-phenanthroline-5,6-dioxime under an inert atmosphere. The reaction mixture was subsequently refluxed for 24 h. The hot reaction mixture was filtered through celite and washed with boiling ethanol. The filtrate was then evaporated under reduced pressure and triturated with 10 mL of water. The mixture was left undisturbed at 4 °C overnight. The formed solid was filtered, washed with additional water, and dried in a vacuum oven at 40 °C to afford the brown product (597 mg, 2.84 mmol) with a 56% yield. The product obtained was used for subsequent steps without any further purification. ^1^H NMR (500 MHz, DMSO-*d*_6_): d 8.77 (dd, *J* = 1.95, 5.2 Hz, 2H), 8.47 (dd, *J* = 2.0, 10.5 Hz, 2H), 7.6 (m, 2H), 5.21 (s, 4H). ^13^C NMR (125 MHz, DMSO-*d*_6_), d 144.9, 140.8, 128.5, 122.7, 122.5, 122.0, 122.0. HRMS (ESI) *m*/*z*: 211.0746, calculated for [M + H^+^] = 211.0978.

##### 2,3-Di(thiophen-2-yl)pyrazino[2,3-*f*][1,10]phenanthroline (**L**)

In a 100 mL round-bottom flask, 200 mg of 5,6-diamino-1,10-phenanthroline (0.95 mmol) and 254 mg (1.2 equiv) of 2,2′-thenil (1.14 mmol) were added, and then 15 mL of ethanol with 5 mL of acetic acid were added together. The reaction mixture was stirred for 6 h at 80 °C, and the yellow precipitate formed in the reaction mixture was filtered, washed with petroleum ether, and dried under a vacuum at 40 °C to give 278 mg (0.70 mmol) of the yellow product with a 74% yield. The precipitate obtained was used in the next step without further purification. ^1^H NMR (500 MHz, CDCl_3_): δ 9.49 (dd, *J* = 8.1, 1.8 Hz, 2H), 9.32 (dd, *J* = 4.6, 1.8 Hz, 2H), 7.83 (m, 2H), 7.58 (dd, *J* = 5.1, 1.2 Hz, 2H), 7.49 (dd, *J* = 3.7, 1.1 Hz, 2H), 7.10 (dd, *J* = 5.1, 3.7 Hz, 2H). ^13^C NMR (125 MHz, CDCl_3_) δ: 165.6, 151.8, 146.1, 141.5, 137.1, 134.1, 129.7, 129.4, 128.0, 127.0, 124.4. HRMS (ESI) *m*/*z*: 397.0599, calculated for [M^+^] = 397.0403.

##### 11,12-Dimethyldipyrido[3,2-*a*:2′,3′-*c*]phenazine (**L′**)

1,10-Phenanthroline-5,6-dione (800 mg, 3.8 mmol) and 4,5-dimethyl-*o*-phenylenediamine (0.517 g, 3.8 mmol) were dissolved in absolute ethanol (300 mL) and refluxed to give a yellow/orange solution. The mixture was refluxed for 2 h with vigorous stirring. The resulting solution was cooled to room temperature, and the white precipitate obtained was filtered, washed with cold ethanol, and dried in a vacuum oven at 40 °C overnight to give 890 mg of a white fluffy solid with a 77% yield. ^1^H NMR (500 MHz, CDCl_3_): δ 9.57 (d, *J* = 8.1 Hz, 2H), 9.24 (m, 2H), 8.02 (s, 2H), 7.76 (dd, *J* = 8.1, 3.6 Hz, 2H), 2.57 (s, 6H). ^13^C NMR (125 MHz, CDCl_3_) δ: 152.2, 148.2, 141.9, 141.7, 140.4, 133.6, 128.3, 127.9, 124.1, 20.7. HRMS (ESI) *m*/*z*: 311.1279, calculated for [M + H^+^] = 311.1291.

### Complex Synthesis

#### Conventional Synthesis of Bi_2_Cl_6_(L)_2_ (**1**)

An oven-dried, two-neck round-bottom flask under a positive pressure of nitrogen was charged with BiCl_3_ (79.5 mg, 0.25 mmol), a ligand (100 mg, 0.25 mmol), and 50 mL of anhydrous acetonitrile. The reaction mixture was heated under reflux with constant stirring for 48 h in a nitrogen atmosphere. After the reaction, the resulting faint-yellow-colored crystalline solid was filtered and washed with diethyl ether and dried in a vacuum oven at 40 °C overnight to give 150 mg (0.105 mmol) of the product with a 41% yield. The complex was used without further purification. The single crystals suitable for structure determination were obtained from the slow evaporation of THF. Anal. calculated for C_44_H_24_Bi_2_Cl_6_N_8_S_4_: H, 1.70; C, 37.12; N, 7.87. Found: H, 1.57; C, 36.92; N, 7.77%. ^1^H NMR (600 MHz, DMF-*d*_7_): δ 9.70 (d, *J* = 2.9 Hz, 4H), 9.61 (d, *J* = 8.0 Hz, 4H), 8.22 (dd, *J* = 4.68 Hz, 4H), 7.98 (d, *J* = 4.0 Hz, 4H), 7.60 (d, *J* = 3.7 Hz, 4H), 7.26 (t, *J* = 5.0 Hz, 4H). Due to the weak signals, ^13^C NMR data could not be collected.

#### Conventional Synthesis of Bi_2_Br_6_(L)_2_ (**2**)

An oven-dried, two-neck round-bottom flask under a positive pressure of nitrogen was charged with BiBr_3_ (84.8 mg, 0.18 mmol), a ligand (75 mg, 0.18 mmol), and 50 mL of anhydrous acetonitrile. The reaction mixture was heated under reflux with constant stirring for 48 h in a nitrogen atmosphere. After the reaction, the resulting dark-yellow-colored crystalline solid was filtered and washed with diethyl ether and dried in a vacuum oven at 40 °C overnight to give 130 mg (0.076 mmol) of the product with a 40% yield. The complex was used without further purification. Anal. calculated for C_44_H_24_Bi_2_Br_6_N_8_S_4_: H, 1.43; C, 31.26; N, 6.63. Found: H, 1.49; C, 34.46; N, 6.20%. ^1^H NMR (400 MHz, DMSO-*d*_6_): δ 9.38 (d, *J* = 8.0 Hz, 4H,), 9.28 (s, 4H), 8.0 (dd, *J* = 3.8 Hz, 4H), 7.89 (d, *J* = 5.0 Hz, 4H), 7.46 (d, *J* = 3.6 Hz, 4H), 7.20 (t, *J* = 4.4 Hz, 4H). Due to the weak signals, ^13^C NMR data could not be collected.

#### Solvothermal Synthesis

##### Bi_2_Br_6_(L)_2_ (**2**)

BiBr_3_ (56.5 mg, 0.12 mmol), a ligand (50 mg, 0.12 mmol), and 20 mL of anhydrous acetonitrile were loaded into a 100 mL Teflon-lined stainless-steel autoclave. The autoclave was sealed and heated slowly over 6 h to 150 °C and then kept at that temperature for 120 h. The oven was cooled to room temperature (35 °C) over 120 h. After the completion of the reaction, the dark, yellow-colored small crystals suitable for the structural determination were obtained and washed with diethyl ether. The 88 mg (0.052 mmol) of the product was isolated with a 41% yield. ^1^H NMR (600 MHz, DMF-*d*_7_): δ 9.75 (s, 4H), 9.67 (d, *J* = 6.8 Hz, 4H), 8.25 (dd, *J* = 4.6 Hz, 4H), 7.99 (d, *J* = 5.0 Hz, 4H), 7.62 (d, *J* = 3.7 Hz, 4H), 7.27 (t, *J* = 4.8 Hz, 4H). Due to the weak signals, ^13^C NMR data could not be collected.

#### Conventional Synthesis

##### Bi_2_I_6_(L)_2_ (**3**)

An oven-dried, two-neck round-bottom flask under a positive pressure of nitrogen was charged with BiI_3_ (148.73 mg, 0.25 mmol), a ligand (100 mg, 0.25 mmol), and 100 mL of anhydrous THF. The reaction mixture was stirred at room temperature for 48 h under a nitrogen atmosphere. After the reaction, the resulting orange-yellow precipitate obtained was filtered, and the filtrate was concentrated under reduced pressure to obtain an orange powdered complex. The complex was further dried in a vacuum oven at 40 °C overnight to give 176 mg (0.089 mmol) of the product with a 35% yield. Anal. calculated for C_44_H_24_Bi_2_I_6_N_8_S_4_: H, 1.23; C, 26.79; N, 5.68. Found: H, 1.35; C, 27.55; N, 4.97%. ^1^H NMR (600 MHz, DMF-*d*_7_): δ 9.81 (s, 4H), 9.72 (d, *J* = 7.9 Hz, 4H), 8.26 (dd, *J* = 4.6 Hz, 4H), 7.99 (d, *J* = 5.0 Hz, 4H), 7.62 (d, *J* = 3.6 Hz, 4H), 7.27 (t, *J* = 4.6 Hz, 4H). Due to the weak signals, ^13^C NMR data could not be collected.

#### Solvothermal Synthesis

##### Bi_2_I_6_(L)_2_ (**3**)

BiI_3_ (74.3 mg, 0.12 mmol), a ligand (50 mg, 0.12 mmol), and 20 mL of anhydrous acetonitrile were loaded into a 100 mL Teflon-lined stainless-steel autoclave. The autoclave was sealed and heated slowly over 6 h to 150 °C and then kept at that temperature for 120 h. Afterward, the oven was slowly cooled to room temperature (35 °C) over 120 h. After reaction completion, the orange-colored small crystals suitable for the structural determination were obtained and washed with diethyl ether. The complex was further dried in a vacuum oven at 40 °C overnight to give 90 mg (0.045 mmol) of the product with a 36% yield. ^1^H NMR (400 MHz, DMSO-*d*_6_): δ 9.39 (d, *J* = 8.4 Hz, 4H), 9.30 (s, 4H), 8.80 (dd, *J* = 4.1 Hz, 4H), 7.89 (d, *J* = 5.0 Hz, 4H), 7.47 (d, *J* = 4.0 Hz, 4H), 7.20 (t, *J* = 3.9 Hz, 4H). Due to the weak signals, ^13^C NMR data could not be collected.

#### Solvothermal Synthesis

##### Bi_2_I_6_(L′)_2_ (**4**)

The reaction was carried out by using the solvothermal synthesis method. BiI_3_ (57 mg, 1 equiv, 0.81 mmol), a ligand (30 mg, 1 equiv, 0.81 mmol), and 9 mL anhydrous acetonitrile were loaded into a 25 mL Teflon-lined stainless-steel autoclave. The autoclave was sealed and heated slowly over 6 h to 150 °C and then kept at the same temperature for 120 h. The oven was cooled slowly to room temperature (35 °C) over 120 h. After the reaction was completed, the orange-colored small crystals settled in the reaction mixture and were filtered off and washed with diethyl ether. The complex was further dried in a vacuum oven at 40 °C overnight to give 60 mg (0.033 mmol) of the product with a 34% yield. Anal. calculated for C_44_H_24_Bi_2_I_6_N_8_S_4_: H, 1.57; C, 26.69; N, 6.22. Found: H, 1.43; C, 27.14; N, 5.69%. ^1^H NMR (600 MHz, DMF-*d*_7_): δ 9.8 (dd, *J* = 8.1, 1.7 Hz, 4H), 9.80 (d, *J* = 4.8 Hz, 4H), 8.24 (dd, *J* = 8.1, 4.7 Hz, 4H), 8.19 (s, 4H), 2.67 (s, 12H).

## Results and Discussion

### General Synthesis Details

The synthesis and characterization of bismuth halide-based coordination complexes is a difficult process due to the stereochemical activity at the bismuth center, which causes the formation of various complexes with different coordination geometries in a single reaction. In this work, we have designed a series of bismuth halide (BiCl_3_, BiBr_3_, BiI_3_)-based coordination complexes with an organic ligand in such a way that the absorption of the complexes could be extended to the visible region of the spectrum and complexes can be studied photophysically. Therefore, to achieve the same, we incorporated the thiophene rings to a highly coordinating bidentate 1,10-phenanthroline ligand^52,53^ and coordinated the organic ligand to different bismuth halides.

The title compounds **1**, **2**, and **3** were synthesized through conventional heating and solvothermal synthesis. As reported in the literature,^22,23,25,27^ we found that small organic ligand-based Bi complexes can be readily synthesized through solvothermal synthesis; however, the stereochemical activity of the bismuth center leads to the formation of phase impure products (Figures S21–S23). The stereochemical activity of the lone pair led to the flexible coordination geometry in Bi^3+^, which caused the formation of monometallic and solvated complexes. Such behavior was predominant when the reactions were performed via the solvothermal synthesis as these reactions were difficult to control, leading to the formation of a mixture of products. On the other hand, conventional heating and stirring proved to be successful in order to harness phase pure bioctahedral bismuth complexes. Finally, we decided to synthesize the complexes via a conventional method, and the solvothermal method proved more successful in yielding crystals of the complexes of sufficient quality for structural determination, which has been proven in the past to be rather challenging. However, we observed that the cooling process during the solvothermal synthesis was the key step in controlling the quality of the single crystals obtained from this method. Quick cooling of the reaction mixture from 150 to 35 °C in ACN in 6 h led to the formation of small poor-quality crystals unsuitable for diffraction. In contrast, when the cooling process was undertaken over 5 days, sizable crystals, suitable for single-crystal X-ray diffraction (SCXRD), were obtained. Additionally, we also managed to grow the single crystals for complexes **1** and **3** through the conventional methods of slow solvent evaporation, solvent diffusion, and vapor diffusion, while for complex **2**, we failed to prepare the crystals for the SCXRD through the conventional methods.

Finally, we found that the choice of the solvent and the ratio of the ligand with respect to the inorganic salt play a dominant role in controlling the reactivity of the bismuth halides to synthesize bismuth halide-based coordination complexes. The correct ratio of the ligand to the bismuth salt played a crucial role in getting the bioctahedral products. An excess of the ligand with respect to the inorganic part can lead to the formation of monometallic complexes with disubstituted bismuth, which leads to the formation of 7-coordinated complexes. Also, as per our experience and the available literature, during the optimization, we observed that acetonitrile and tetrahydrofuran favored the formation of the bioctahedral products with minute impurities, while when the same reactions were attempted in other solvents, excess of solvated products were observed.

### NMR Spectroscopy

**Figure 2:**
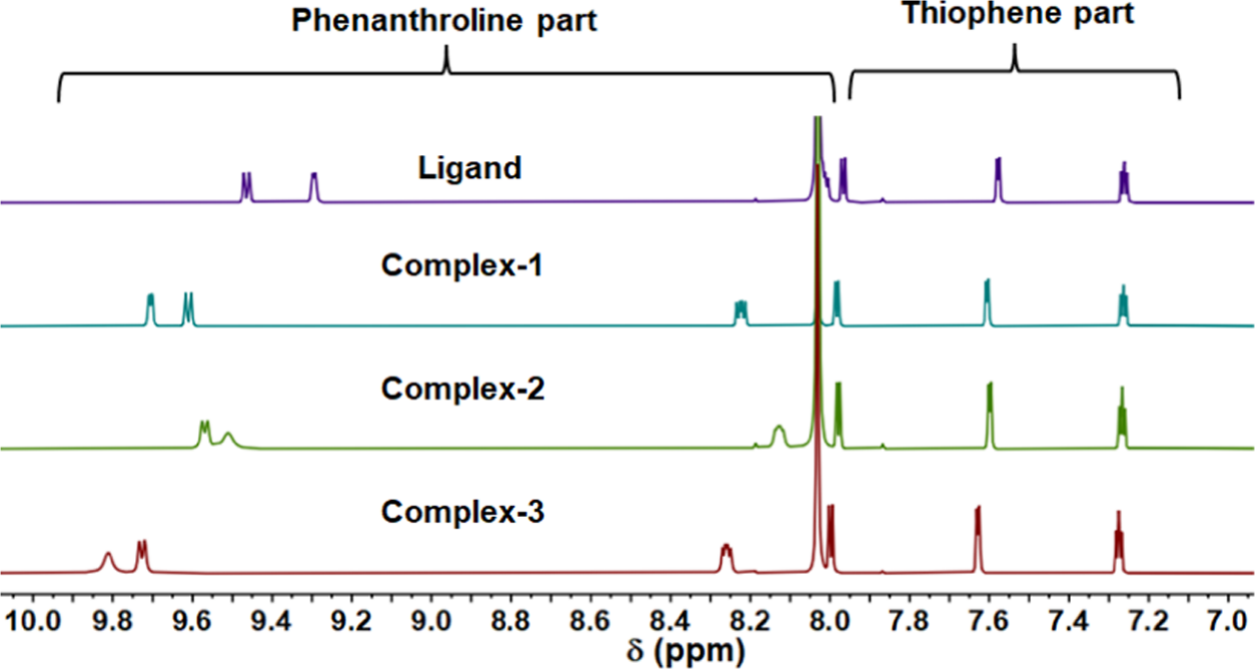
Comparison of ^1^H NMR (600 MHz) spectra of the ligand and complexes in DMF-*d*_7_.

To confirm the differences in molecular structures as well as the long-term stability of the complexes in solution, ^1^H NMR spectroscopy was recorded in DMF-*d*_7_ over a period of 7 days, as shown in Figures 2 and S7–S12.^54^ Additionally, the kinetics studies proved that all complexes do not degrade in the solution and hence remain stable for a period of 7 days. The spectra of all of the complexes were further compared with the ligand (**L**) to understand the electronic effect of the presence of metal and a halide ancillary ligand over the proton resonances in the complexes (Figure 2). The proton NMR spectrum of **L** consists of six resonance peaks at 9.45, 9.29, 8.00, 7.96, 7.57, and 7.25 ppm, as expected from the rigid symmetric molecule. The ^1^H NMR spectra of all of the complexes show single resonance peaks for each of the associated protons, which further confirms the magnetic equivalence and high symmetry of the complexes in solution. However, the formation of coordination bonds of **L** with different bismuth halides leads to significant downfield shifts in the phenanthroline proton resonances compared with **L** (Figure 2). The proton resonances for the phenanthroline part of the complexes shift to 9.70, 9.61, and 8.22 ppm in **1**; 9.74, 9.67, and 8.24 ppm in **2**; and 9.81, 9.72, and 8.25 ppm in **3**, while the thiophene ring resonances are largely unaffected. Comparatively, the proton resonances of complex **3** are more deshielded than **1** and **2**, supporting the idea of differences in Lewis acidity of the bismuth center.^55^

### Crystallography

**Figure 3:**
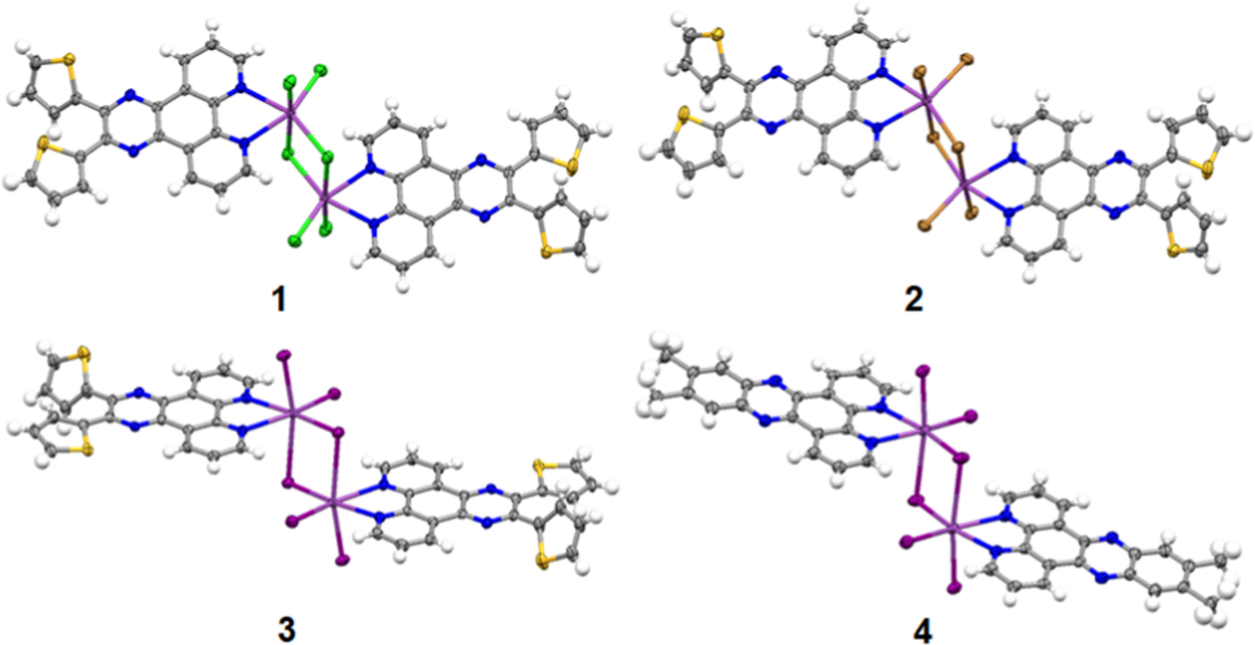
Perspective views of complexes **1**, **2**, **3**, and **4**, as determined by single-crystal X-ray diffraction.

X-ray crystallographic analysis of compounds **1**–**3** was carried out to investigate the coordination geometry and molecular packing of each coordination compound. Single crystals of complex **1** were obtained from the slow evaporation of THF, while for complex **2**, the crystals were obtained solely from the solvothermal synthesis. The crystals for complex **3** were obtained through various methods, including solvothermal synthesis, vapor diffusion of ethanol to DMF, and slow evaporation of a THF solution of the complex. All complexes crystallized in a bioctahedron coordination geometry with each molecular unit consisting of two Bi(III) centers, each coordinated to a thiophene-substituted phenanthroline ligand (**L**) along with chlorine, bromine, and iodine as the ancillary ligands in **1**, **2**, and **3**, respectively (Figure 3). Each bismuth atom in a molecule coordinates with one organic ligand and four units of halogens, while two bismuth units in a molecule are bridged by two units of halogens (Cl, Br, I).

All of the complexes (**1**–**3**) are packed with four molecules in the unit cell. Complexes **1** and **2** crystallized in an orthorhombic crystal system with a *Pbca* space group, while complex **3** crystallized in a monoclinic crystal system with a *C*2/*c* space group (Table S1).

The Bi–N_L_ bond distances in **1** are 2.472(2) and 2.506(2) Å, and the Bi–Cl bond distances range from 2.512(7) to 2.900(8) Å. The distance between the two bismuth centers in a molecule is 4.134(6) Å (Figure S17a). In case of **2**, the Bi–N_L_ bond distances are 2.507(2) and 2.528(2) Å, the Bi–Br bond distances range from 2.652(5) to 3.061(5) Å, while the distance between the two bismuth centers in a molecule is 4.274(6) Å (Figure S17b). Complex **3** consists of comparatively elongated bonds, as shown in Figure S17c. The Bi–N_L_ bond distances are 2.559(5) and 2.601(5) Å, while the Bi–I bond distances range from 2.864(5) to 3.361(5) Å. The distance between the two bismuth centers is observed to be 4.449(8) Å. The change of the bond distances with the change in the ancillary ligand is illustrated in Table S2, and the comparison of the data suggests that the change in the size of the halide in the complexes weakens the Bi–N_L_ bond, thus increasing the bond length. In all of the cases, the bridged bismuth–halogen bond distances are longer as compared to that of unbridged bismuth–halogen bonds, which further elongates the distance between the two bismuth center atoms from **1** to **3**. Similar differences in bond distances of bridged and unbridged ligands along with the Bi–N bonds have been observed previously.^56−59^ Moreover, the increase in the size of the bonding orbital from a Cl and Br to I-based ancillary ligand further suggests the weak bismuth–halide bonds in **3** as compared to that in **1** and **2**. In **1** and **2**, both the thiophene rings face each other with dihedral angles of −42.8(3) and 164.2(2)° and −46.0(3), and 161.5(2)° between the atoms N_4_–C_14A_–C_19A_–S_2A_ and N_3_–C_13A_–C_15A_–S_1A_, respectively (Figure S18). Interestingly, in **3**, both the rings oppose each other and face toward the pyrazine moiety with the dihedral angles of 53.1(6) and 10.9(5)° when calculated along N_4_–C_14_–C_19A_–S_2A_ and N_3_–C_13A_–C_15A_–S_1A_, respectively (Figure S18). The distance between the nitrogen atoms of a pyrazine moiety and the sulfur atoms is observed to be 3.082(2) and 3.970(2) Å in **1**, 3.127(3) and 3.967(3) Å in **2**, while 2.760(5) Å and 3.127(6) Å in **3** (Figure S18). Such a difference in orientation and distance of the thiophene rings in **3** when compared with **1** and **2** is indicative of a stronger electronic push effect from the electron-rich thiophene moiety toward that electron-deficient pyrazine ring.

Moderate to weak π–π interactions exist among the ligands of all complexes. In complex **1**, weak π–π interactions (centroid to centroid) of 3.815 and 3.868 Å exist between the phenyl rings of the phenanthroline part and pyrazine rings, respectively, along with the strong interaction of 3.324(2) Å between C_16A_=C_15A_···C_17A_=C_18A_ (Figure S19). Whereas in the case of **2**, a similar weak interaction of 3.752 Å exists between the centroid of the phenyl and pyrazine rings along with a stronger π···π interaction of 3.276(2) Å between C_16A_=C_15A_···C_17A_=C_18A_. Surprisingly, in the case of **3**, comparatively stronger π···π interactions are present between the ligands with the distance of 3.552, 3.697, and 3.810 Å between the centroid of the pyrazine, thiophene, and phenanthroline rings, respectively (Figure S19). Additionally, weak n–π interaction is present at a distance of 3.489(6) Å between the free lone pair of electrons of S_1A_ and C_1_=C_2_ along with stronger lp(S_2A_)···lp(S_2A_) interactions at a distance of 3.171(8) Å (Figure S20). Additionally, the PXRD data for the crystalline complexes were collected to analyze the purity of each batch of complexes that were obtained through the reaction carried out by the conventional synthesis (Figures S21–S23). In the case of **1** and **2**, minor crystalline impurities were identified in the bulk when using the solvothermal synthesis. However, for compound **3**, the disparities observed in the PXRD spectra were more pronounced, indicating the creation of a physical mixture. This mixture comprises the structurally characterized crystal form of compound **3** along with a significant quantity of an unidentifiable crystalline phase, which could potentially be a polymorph or a solvate of compound **3**.

### UV–Visible Absorption Spectroscopy in Solution

**Figure 4:**
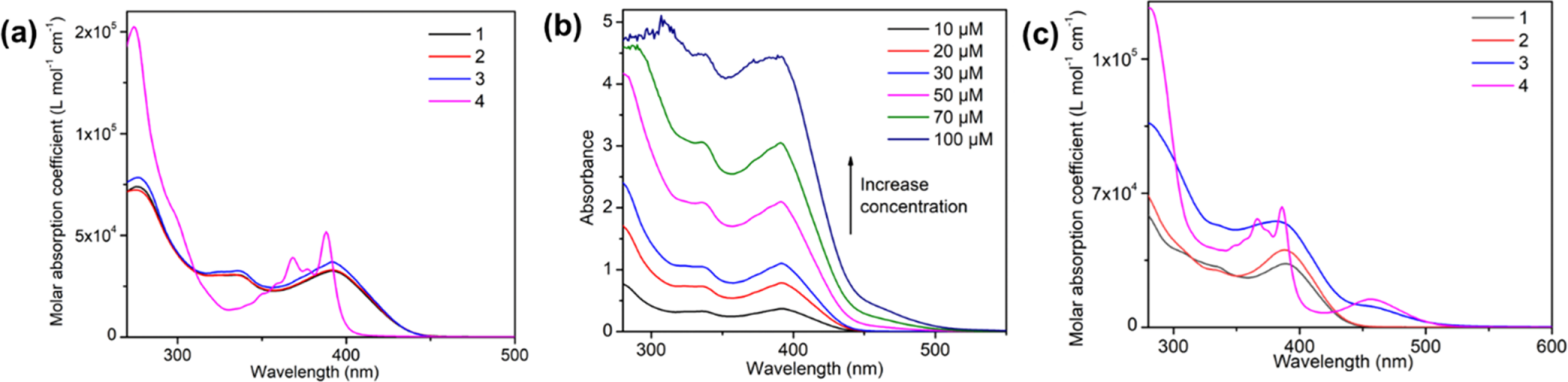
(a) Absorption spectra of **1**–**4** in DMF at 10 μmol, (b) concentration-dependent absorption spectra of **3** in DMF, and (c) comparison of absorption spectra of **1**–**4** in ACN at 10 μmol.

The ground-state absorption spectra of the ligand **L** and the metal complexes **1**, **2**, and **3** were recorded in dimethylformamide (DMF) and acetonitrile (ACN) at a concentration of 10 μM, as shown in Figure 4. The data for the absorption spectra are summarized in Table 1 and Tables S3–S4. Additionally, we recorded the absorption spectra of BiCl_3_, BiBr_3_, and BiI_3_ at a concentration of 10 μM in DMF and ACN (Figure S24).

**Table 1:** Photophysical Properties of Complexes 1, 2, and 3 in DMF

	λ_abs_, nm	ε, M^–1^cm^–1^	λ_em_, nm	τ_Fl_, ns	Φ_Fl_ %
1	392	31 100	467	0.32(±0.06) (23.1%),	3.3
	335			0.47(±0.02) (76.9%)	
2	392	38 900	468	0.38(±0.03) (67.6%),	2.2
	335			0.53(±0.06) (32.4%)	
3	392	45 700	467	0.36(±0.04) (49.8%),	4.5
	335			0.51(±0.04) (50.2%)	

By comparing the absorption intensity of all complexes in DMF, the molar absorption coefficient ε at ∼392 nm was maximum in complex **3** (ε_392_ ∼ 45,700 M^–1^ cm^–1^), while the intensity decreased as the size of the ancillary ligand reduced from Br (ε_392_ ∼ 38,900 M^–1^ cm^–1^) to Cl (ε_392_ ∼ 31,100 M^–1^ cm^–1^) (Figure 4). As expected, the absorption coefficient at 392 nm was much lower in the ligand (ε_392_ ∼ 13,200 M^–1^ cm^–1^). The difference in the absorption intensity at a lower-energy level for the complexes as compared to the ligand confirms the complexation of the bismuth salt with the ligand. Moreover, the change in the absorption coefficient for the different complexes in DMF further suggests the contribution of ancillary ligands in controlling the ground-state absorption. The absorption spectra of complexes **1**–**3** and **L** are similar to each other, and all exhibit similar absorption bands in the near-UV and visible region. The intense absorption band at ∼285 nm in the complexes and ligand corresponds to the π–π* transition from the phenanthroline part of the ligand to the higher energy states of the ligand and the complexes (Figure 4, S25), while the lowest energy absorption band at ∼392 nm in complexes and the ligand overlaps together, thus suggesting that this absorption band corresponds to the singlet ligand-centered (^1^LC) transition related to the charge transfer from the thiophene rings to the more electron-deficient phenanthroline ring. In the case of the bismuth salts in DMF, a highly intense absorption band was observed between 260 and 275 nm, and a lower-energy band was observed for BiBr_3_ and BiI_3_ at 375 nm along with a weak absorbing tail for BiCl_3_ (350–410 nm) (Figure S24). As reported,^60−62^ the halide complexes of bismuth are known to show two types of electronic bands, metal-centered (MC) sp bands and halogen to metal-centered LMCT bands. Generally, the LMCT transitions are observed to be higher in energy; however, upon changing the halides coordinated with a bismuth atom from Cl and Br to I, the contribution of a higher energy LMCT transition shifts toward the lower-energy side and its contribution increases in sp absorption bands (Figure S24). Therefore, comparing the lower-energy absorption bands (∼392 nm) of the complexes with the salts confirms that the absorption band in the complexes consists of multiple overlapping electronic transitions due to the ^1^LC state (phenanthroline–thiophene ligand), the halogen to metal center ^1^LMCT band, and the metal-centered, ^1^MC, band (Figures S26–S27).

To understand the effect of concentration on the ground-state absorption, concentration-dependent (10–100 μM) absorption studies were performed for complexes **1**–**3** and **L** (Figure 4, figure S28) in DMF under ambient conditions. For compounds **1**, **2**, and **L**, an increase in absorption intensity at 392 nm was observed with no additional absorption band at the lower energies, thus suggesting the absence of intermolecular π–π interactions in the complexes and the ligand. However, for complex **3**, an increase in concentration leads to the significant appearance of a lower-energy absorption band between 450 and 550 nm (Figure 4), which otherwise is weakly observed at a concentration of 10 μM. Such a lower-energy absorption band in **3** initially suggests the role of intermolecular π–π interactions in affecting the ground-state absorption. However, to further confirm the origin of the lower-energy absorption band, the absorption spectra of BiI_3_ at different concentrations in DMF (10–100 μM) were recorded (Figure S28). As shown in Figure S28, with increasing concentration, a new lower-energy absorption band appears and extends to ∼550 nm. The observation of the new band and its extension to 550 nm in BiI_3_ is caused by the mixture of an interconfigurational transition from 6s^2^ to 6s^1^ 6p^1^ along with the LMCT transition from the σ orbital of iodine to the *p* orbital of bismuth.^60,63^ Therefore, the observation of the lower-energy band at 450–500 nm of **3** in DMF can be clearly assigned to transitions taking place in the inorganic part of the complex. Whereas in the case of **1** and **2**, no lower-energy band extending up to ∼550 nm is observed due to the comparatively weaker electron-donating tendency of the chlorine- and bromine-based complexes, hence weaker electronic transitions from halogens are involved in the **1** and **2**. However, the absorption spectrum of BiBr_3_ shows a lower-energy band (Figure S24) between 345 and 410 nm, which corresponds to the mixture of LMCT (σ orbital of bromine to the *p* orbital of the bismuth atom) and sp transition, while in BiCl_3_, only the tail of lower-energy interconfigurational sp transition is observed.^64^

To further confirm the observations in complex **3**, we designed and synthesized a new complex **4** (Figure 1) with weaker electron-donating methyl groups attached to phenanthroline. The absorption studies for complex **4** at different concentrations (10–100 μM) were repeated in DMF (Figure S30). Similar to complex **3**, a new weakly absorbing broad band was observed between 410 and 500 nm for complex **4** (Figure 4, Figure S29) at a lower concentration, but with an increase in concentration, the intensity of the absorption increased. The comparison of absorption spectra between complexes **3**, **4**, and BiI_3_ (Figure S31) shows that a lower-energy band is observed in both iodo compounds, thus confirming that the lower-energy band originates from the inorganic BiI_3_ part of the complexes rather than from π–π stacking or similar noncovalent interactions. Finally, the absorption studies of **1**, **2**, **3**, and **4** confirm that the lower-energy band at 392 nm is composed of multiple transitions involving different parts of the complexes. In complex **1**, the absorption at 392 nm consists of a metal-centered sp transition, an intraorganic ligand transition, and a transition between the metal and ligand, while in complex **3**, the lower-energy absorption band mainly involves transitions from the bismuth center (sp), weak LMCT (halogen to metal), intraorganic ligand transition, and transition between the metal and ligand. In complexes **3** and **4**, the absorption bands at 392 and 410–500 nm are due to the transition of the organic ligand, the bismuth atom, between the metal and organic ligand, and strongly from the ancillary ligand iodine to the bismuth center. The comparative studies of all of the complexes with the inorganic salts also confirm that the ground-state electronic properties are strongly influenced by the inorganic part in bioctahedral-based coordination complexes.

To further understand the solvent-dependent properties of the complexes, we recorded the absorption spectra for the complexes in ACN at 10 μM. As shown in Figure S25c, the change in the solvent polarity led to a blue shift in the absorption bands. The lower-energy absorption band shifts to 388 nm for **1** and **2** and to 382 nm for complex **3**. Additionally, complex **3** consists of a lower-energy absorption band (440–525 nm) at 10 μM, while this lower-energy band is absent in complexes **1** and **2**. The origin of the higher (382–388 nm) and lower-energy bands (440–525 nm) corresponds to the same transitions as observed for DMF when compared with the absorption spectra of the inorganic salts (Figure S25). Therefore, the observation of a band at ∼440 to 525 nm in both solvents suggests the origin of similar species. Finally, from the above studies, we can conclude that the lowest energy band corresponds to the halide–metal LMCT transition.

Combining the results of the detailed absorption studies on **1**–**4** proves that BiI_3_-containing coordination complexes **3** and **4** absorb strongly in the visible region due to the presence of multiple transitions associated with the organic ligand, iodine, and metal center due to the presence of strong electron-donating and highly diffused σ orbitals on the iodine. In the case of **1** and **2**, such lower-energy bands in the visible region are absent due to the poor electron-donating σ orbitals. Therefore, the change in the ancillary ligand from chloride to bromide in bioctahedral bismuth complexes causes the enhancement of the oscillator strength of lower-energy absorption transitions, while for iodine, in addition to the increase in the absorption coefficient, a new lower-energy absorption band in the visible region is observed. To further understand the effect of the ancillary halide ligands on the electronic structure, we recorded the ultraviolet photoelectron spectra (Figures S43–S45). Complex **1** displayed the highest work function of 4.9 eV, whereas the work functions for complexes **2** and **3** were slightly lower at 4.2 and 4.4 eV, respectively.

#### Steady-State Emission Spectra in Solution

**Figure 5:**
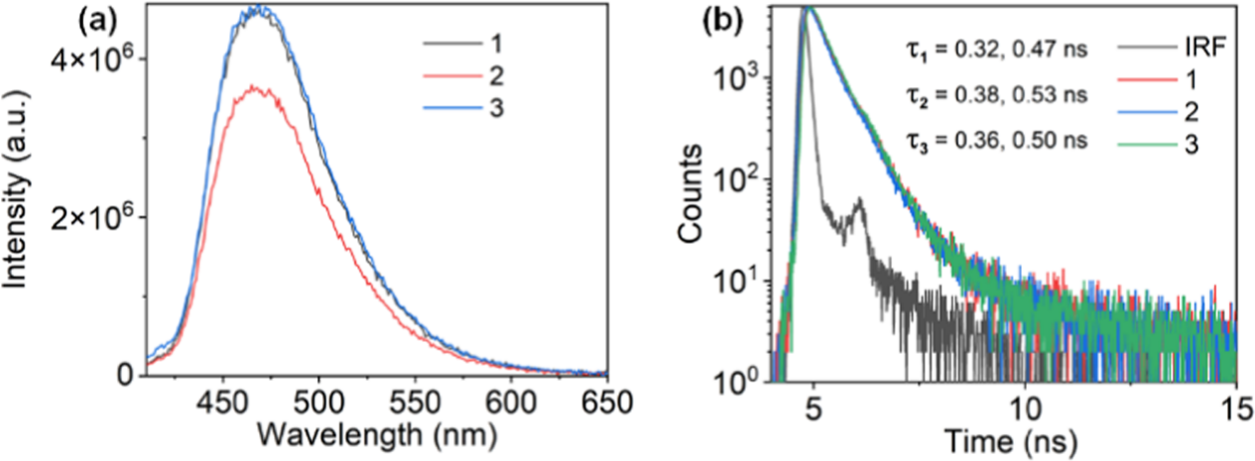
(a) Emission spectra and (b) lifetime of complexes **1**–**3** in DMF (10 μmol) at excitation of 392 nm.

In order to understand the excited-state behavior of the complexes in different solvents, photoluminescence and time-resolved studies were performed. The photoluminescence spectra of all of the complexes were recorded in THF, ACN, and DMF at excitation of 392 nm (concentration = 10 μM), as shown in Figures 5 and S32–S33, Tables S3–S4. The emission maxima of the complexes show bathochromic shifts from 461 nm in THF to 467 nm in ACN and DMF. The small 6 nm red shift in emission with increasing solvent polarity suggests that the excited state shows weak charge transfer in nature (Figure S32 and S33). The comparison of the emission spectra of all of the complexes with the ligand shows that the emission of ligand superimposes that of **1**–**3** (Figure S33), thus confirming that the lower-energy emissive excited state in the complexes is ligand-centered (^1^LC). Additionally, the charge transfer feature observed in the complexes from changing the solvent polarity is assigned to the electron-donating strength of the thiophene rings attached to the phenanthroline. The lifetime of the complexes and ligands was measured with a 375.6 nm picosecond pulsed laser diode at the emission maxima. As shown (Figure 5, S34, Tables 1, S3, and S4), **1**–**3** decay radiatively following the biexponential decay in all solvents. The radiative decay of **L** monitored at 467 nm in DMF shows lifetimes of 0.39(±0.01) and 0.61(±0.05) ns, while in complexes **1**–**3**, the lifetimes were observed to be 0.32(±0.06) and 0.47(±0.02) ns for **1**, 0.38(±0.03) and 0.53(±0.06) ns for **2**, and 0.36(±0.04) and 0.51(±0.04) ns for **3**, respectively The observation of similar lifetimes for the ligand and complexes further confirms the ^1^LC nature of the excited state. However, when the lifetimes of the complexes are compared in all solvents, the decay pattern for the complexes changes with the solvent polarity and is observed to decay fast in ACN (Figure S34, Tables 1, S3, and S4).

The comparison of emission intensity and photoluminescence quantum yield of **1**–**3** in THF, ACN, and DMF (Figure S33 a,d,e, Table 1, S3 and S4) shows that all of the complexes emit weakly in ACN, while the intensity of emission remains relatively more for THF and DMF. Such a difference in the emission behavior of the complexes and the ligand in ACN when compared with other solvents suggests the varied behavior of the excited state of these complexes in solvents of different polarities. Comparatively, all complexes are relatively strongly emissive in DMF, and such differences in observed quantum yields with the change of the solvent also further imply the effect of the solvent to control the excited-state dynamics of the complexes.

**Figure 6:**
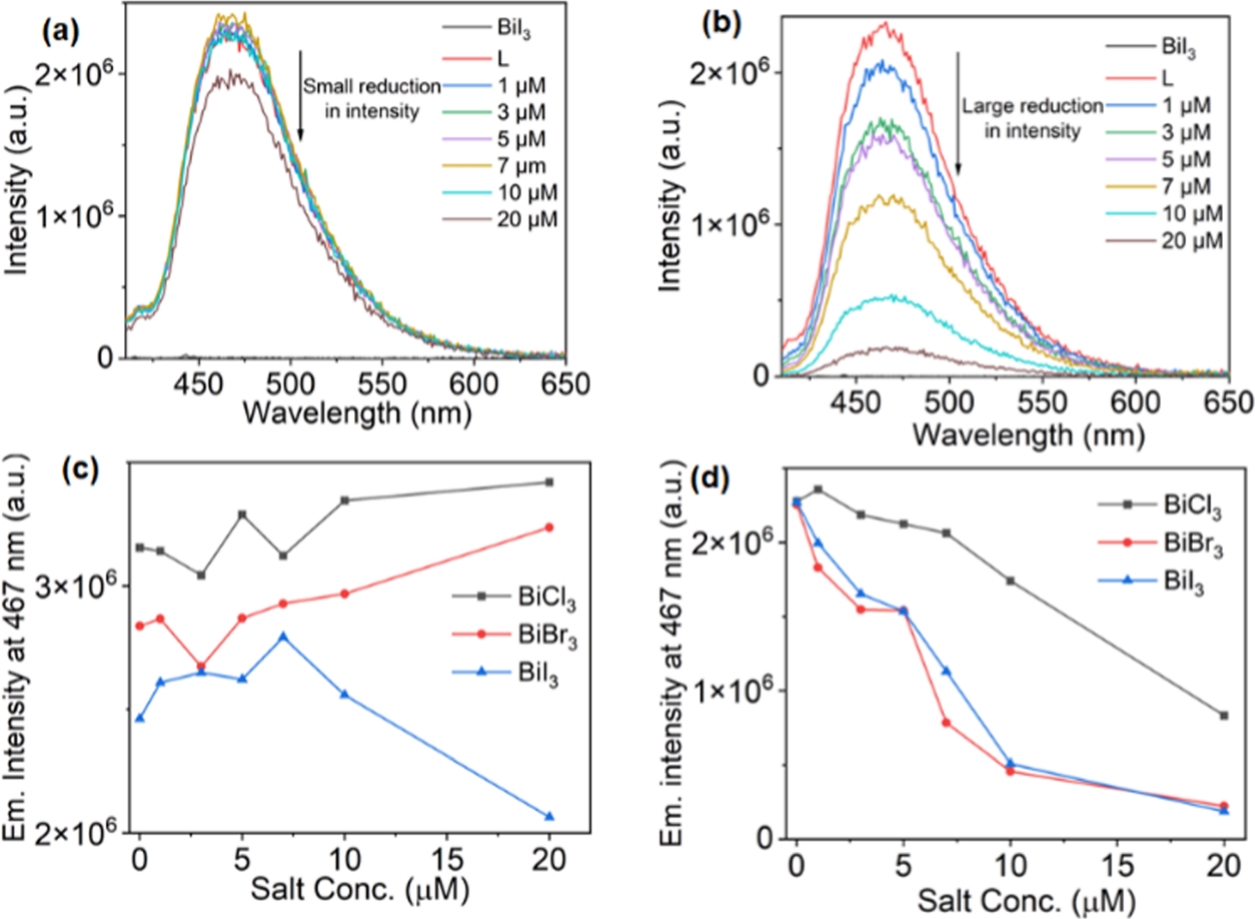
Titration studies of **L** (10 μmol) in (a) DMF and (b) ACN at an excitation of 392 nm with BiI_3_. The comparison of the change in the emission intensity of **L** with the slow addition of BiCl_3_, BiBr_3_, and BiI_3_ in (c) DMF and (d) ACN.

To further understand this behavior, we performed a titration study between the ligand and the corresponding Bi salt (Figure 6, S35). The concentration of the ligand was kept at 10 μM, while the amount of the inorganic Bi part was increased from 1 to 20 μM in ACN. Initially, the emission spectra of only the ligand and inorganic salt at a 10 μM concentration were recorded using an excitation wavelength of 392 nm. As shown in Figures 6 and S35, all of the salts (BiCl_3_, BiBr_3_, and BiI_3_) emitted negligibly when excited, while the ligand emitted strongly in all cases (graphs **1-Cl, 2-Br**, **3-I**). The slow addition of the inorganic salt to the 10 μM solution of the ligand caused a reduction in emission intensity in all cases. For **1-Cl**, the reduction in emission intensity was relatively weak, and only the addition of excess BiCl_3_ (20 μmol) caused a significant drop in emission intensity of **L**, while at lower concentrations (1–10 μmol), the change in intensity was negligible. By comparison, a significant reduction in emission intensity was observed for **2-Br** and **3-I** at a concentration of 1 μM. The addition of only 1 μM inorganic salt lowered the emission intensity by 18.9 (**2-Br**) and 12.1% (**3-I**), in stark contrast to **1-Cl** for which a small increase of 3.5% was observed. However, the addition of 10 and 20 μmol salts to the ligand causes a large reduction of intensity in all mixtures. Such behavior of the ligand in contact with the free inorganic salts is like that observed in complexes **1**, **2**, and **3** in ACN, as explained earlier. Moreover, the difference in the reduction of emission intensity of the ligand in **1-Cl** when compared with **2-Br** and **3-I** suggests the role of additional factors in quenching the excited state. However, when the same studies were performed in DMF, no significant change in the intensity of the ligand was observed (Figure 6a,c and S35). Such differences in the emission intensity during the titration studies in ACN and DMF suggest the role of additional factors in affecting the excited-state properties of the complexes in different solvents.

#### Photophysical Studies in Aggregates

**Figure 7:**
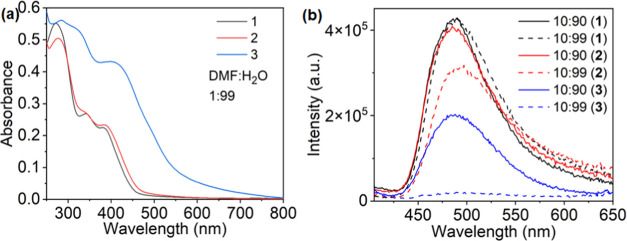
(a) Absorption and (b) emission spectra (excitation wavelength = 392 nm) of **1**–**3** in DMF:H_2_O at a concentration of 10 μmol.

In order to understand the effect of aggregation on the photophysical properties of the complexes, absorption, emission, and lifetime measurements were performed at a 10 μM concentration in the mixture of DMF and water (Figure S36, S37 and S38). Figure S36 shows the absorption spectra of **1**–**3** with an increasing water fraction from 0% to 99%. Initially, complexes **1**–**3** showed a lower-energy absorption band at 392 nm in 100% DMF. The slow increase of the water content of DMF from 0 to 70% led to the reduction in the absorption coefficient of the absorption band at 392 nm in all complexes. Additionally, the slope of the lower-energy absorption band shows a slight shift toward lower energy, as shown in Figure S36. This shift may be attributed to the enhanced π–π interaction of the ligands and the inorganic part of the complexes and increases with the size and π-donation ability of the ancillary ligand increases. The addition of water from 70 to 99% to DMF caused the aggregation of molecules, which led to a further broadening of the absorption band along with the observation of a long tail due to the scattering with the aggregates. Interestingly, at 99% water added, a reduction in absorption intensity, as well as a blue shift (**1**: 392 to 380 nm; **2**: 392 to 382 nm), was observed for the lower-energy absorption band. Moreover, the reduction in the absorption intensity confirmed the reduced oscillator strength of **1** and **2**. In contrast, the addition of 99% water to **3** showed the red shift and broadening of the absorption band along with the enhancement of absorption intensity, which suggests an enhanced oscillator strength because of aggregation in complex **3** (Figure 7). Additionally, a new absorption band at 490 nm was observed due to the enhanced interactions of the σ-orbitals of iodine with the π*-orbital of the ligands during aggregation and the enhanced interaction of the ancillary ligand with the metal center. Moreover, a comparison between the absorption spectrum of **3** at 99% water with the absorption spectra recorded at 100 μM DMF and 10 μM of THF and ACN shows the origin of the same absorption band, thus confirming that the lower-energy absorption band originates due to the high electron-donating tendency of the ancillary ligand, which otherwise is absent in complexes **1** and **2**.

The photoluminescence spectra of all complexes **1**–**3** were also recorded with increasing water content (0–99%) in DMF. As shown in Figure S37, in 100% DMF, all complexes emit at ∼468 nm, suggesting that similar excited states are responsible for the emission. Increasing the water content of DMF from 0 to 70% red shifts all emission bands from 468 to 480 nm. Alongside, a reduction in the emission intensity was observed with increasing water content, which suggested that aggregation-caused quenching (ACQ) as well as other nonradiative pathways are responsible for the reduction in emission intensity.

Further addition of water (70–99%) enhanced the aggregation of the complexes along with a red shift in the emission maxima to 492 nm and the emergence of an additional emission band at ∼575 nm in **1** and **2**. The observation of an additional lower-energy emission band can be ascribed to the packing of the aggregates with the aggregation of the molecules. In comparison, when the water content is increased to 99%, the emission of complex **3** becomes negligible. Such a drastic decrease in emission most likely originates from enhanced aggregation, strong spin–orbit coupling due to the iodine causing nonradiative decay, and the presence of low-energy states. However, the lifetimes observed during aggregation were also found to be similar with increasing water content, and the long lifetimes at (DMF:H_2_O; 1:99), as shown in Table S5, could be due to the scattering from the aggregates. Additionally, comparing the emission intensity of the various aggregates (**1**–**3**) in Figure 7 highlights that upon increasing the water content from 90 to 99%, the emission intensity of **1** is barely affected, while it is markedly reduced in the case of **2** and **3**. This provides further evidence that additional factors, other than ACQ and heavy element effects, play a role in controlling the excited-state dynamics.

Comparing the absorption studies in solution and upon aggregation suggests that the lower-energy transition (450–500 nm) in complex **3** is dependent on the solvent and intermolecular interactions. As described earlier, such a lower-energy band in the iodine-containing complexes originated from the interaction of the ancillary ligand with the metal center; however, the intensity of observation of this band varies with the solvent system used as it is weakly observed in DMF as compared to ACN. In addition, the aggregation studies showed that close interaction of the bioctahedral molecules in the aggregates can also extend the absorption of the molecules to the visible region, hence making such molecular systems suitable for solar light absorption-based applications.

#### Nanosecond-Microsecond Transient Absorption Spectroscopy

**Figure 8:**
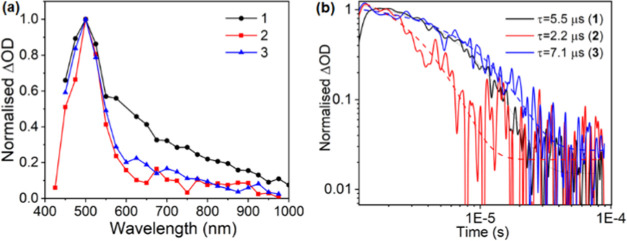
(a) Normalized (to 1) transient absorption spectra of **1−3** in DMF using an excitation pump wavelength of 420 nm and excitation densities of ∼11 μJ cm^−2^. (b) Normalized transient absorption decay dynamics for **1−3** in DMF, pumping at 420 nm with an excitation density of ∼26 μJ cm^−2^ and probing at 500 nm.

To investigate the triplet excited state of the complexes in the solution, we studied the nanosecond-microsecond transient absorption spectra (Figures 8 and S39–S41). The transient absorption spectra of **1**–**3** were collected in deoxygenated DMF using 420 nm as the pump wavelength at different delay times (1–50 μs), following the pump excitation, as shown in Figure 8 and Figures S39–S41. The formation of an excited-state absorption (ESA) band was observed at 500 nm for all of the complexes. The ESA band was quenched in the aerated solution and again returned to the original after deaeration for all complexes, thus confirming that the signal resulted in the formation of a triplet state (Figures S39–S41). The different spectra recorded at the time scale of 1 to 50 μs consist of a single ESA band at 500 nm and were assigned to ^3^LC in all cases. The spectra appear at similar time scales and with similar features for complexes **1**–**3**, further suggesting the origin of a single triplet state species in the complexes. The decay at 500 nm was modeled using a single exponential function, and the lifetime determined from the transient absorption data for the triplet excited state is 5.5, 2.2, and 7.1 μs for **1**, **2**, and **3**, respectively. The difference in the decay time of the triplet state could be due to the varied effects of different inorganic parts in controlling the excited state. No triplet-state emission was observed during the emission measurements in DMF under the experimental conditions, thus indicating that the lowest triplet excited state in the complexes is a dark state. However, the origin of the triplet state at the same position for all complexes and similar lifetimes of the transient state in complexes **1**–**3** further shows that no other side reactions take place in the measured time regime, and only a single excited-state species is involved. This further confirms that the photoredox decomposition of the complexes is absent in such bioctahedral bismuth complexes, which otherwise is normally observed due to the halide ligand to metal charge transfer in bismuth halides.^61,62,64^

### Computational Studies

The crystal structures of compounds **1**–**3** were relaxed by using the PBE+D3 functional to obtain the ground-state geometry. The resultant PBE+D3-optimized lattice parameters are listed in Table 2. Overall, PBE+D3 performs very well at replicating the experimental parameters at 150 K, with all parameters remaining within 1% of the experiment. To demonstrate the effect of the inclusion of van der Waals corrections to the structure, we also include PBEsol for the chloride compound—while it performs similarly to PBE+D3 in two dimensions, it severely overestimates the packing in the crystallographic b direction—predominantly corresponding to the π–π stacking direction within the crystal, demonstrating the importance of dispersion interactions in correctly describing the crystal structures of these complexes.

**Table 2:** Lattice Parameters of Compounds 1–3, with Percentage Differences from the 150 K Experiment (Table S1) Given in Parentheses^a^

system	*a* (Å)	*b* (Å)	*c* (Å)	α (deg)	β (deg)	γ (deg)
1 (PBE+D3)	22.48 (+0.73%)	7.542 (+0.19%)	26.12 (−0.89%)	90	90	90
1 (PBEsol)	21.93 (−1.75%)	8.317 (+10.5%)	26.13 (−0.88%)	90	90	90
2	7.626 (−0.21%)	26.27 (−0.01%)	23.05 (+0.10%)	90	90	90
3	7.993 (+0.28%)	17.57 (−0.26%)	18.32 (+0.43%)	89.22 (+0.05%)	86.59 (+0.24%)	76.85 (−0.09%)

a Compound 3 is given as the corresponding primitive cell due to the removal of crystallographic disorder.

**Figure 9:**
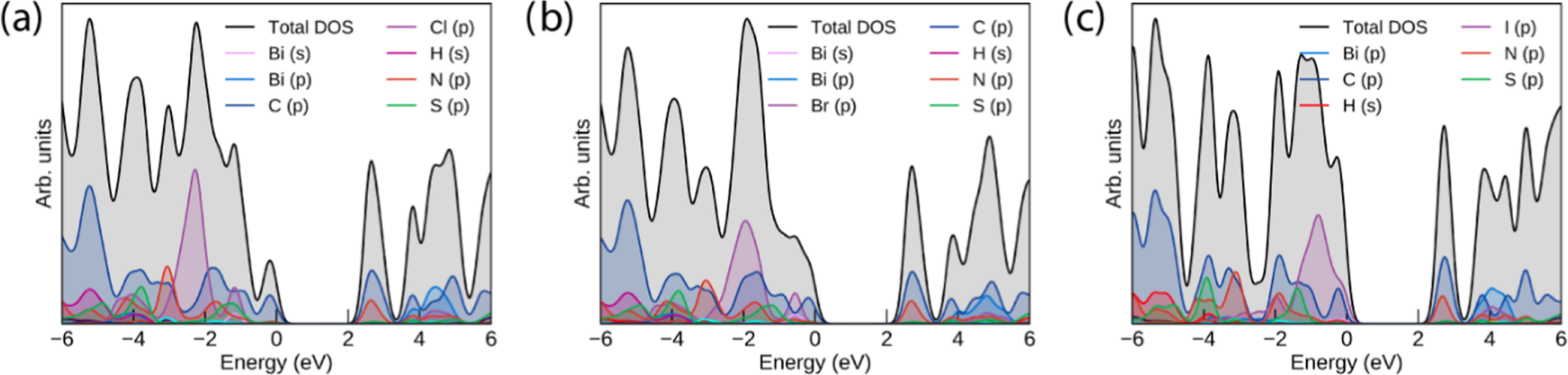
HSE06 density of states (DOS) of compounds **1–3 (a–c)**, plotted using the sumo package.^65^ All densities of states have *E* = 0 eV set to the valence band maximum; a Gaussian smearing of 0.2 eV is applied to all states.

The PBE+D3-optimized structures were used as the basis for electronic structure calculations with the HSE06 and HSE06+SOC methods, which are suitable for determining band gaps in extended complexes with reasonable accuracy. Due to the structurally localized nature of the complexes, there is minimal band dispersion in these systems, with only some in the Bi p-dominated states in the conduction band (and to a lesser extent, the Bi s/I p states in the valence band of compound **3**). As such, the electronic band structure, plotted across the Brillouin zone, is not informative for these systems. The densities of states of the three compounds, however, still help to show the distribution of organic and inorganic states in the solid state, and those calculated with the HSE06 functional are shown in Figure 9.

The HSE06 densities of states show strong similarity across all three compounds in the position and contributions of the organic (C p/N p dominant) and Bi p states, consistent with the identical composition with the exception of the halide but indicating that there is also minimal hybridization between the electronic state in the inorganic and organic sections of the complexes. In compounds **1** and **2**, the frontier states around the gap are dominated by the organic ligand, strongly in-line with the dominance of the ^1^LC transition in the absorption spectra, as shown in Figure 4 a. The partial charge density plots of the valence band maximum (VBM) and the conduction band minimum (CBM) of compound **1** in Figure S42 further demonstrate the earlier assignment that the transition specifically arises from the charge transfer from the thiophene rings to the phenanthroline section involved in binding to the bismuth.

As halide changes from Cl to I, the halide p states follow the expected trend of increasing energy with respect to the organic levels; in the iodide compound, this is sufficient for them to equally dominate the valence band maximum. In all three compounds, the halide p bands are split into two sets: the lower-lying states that are purely halide-dominated and the higher-lying bands that involve the antibonding contribution from mixing with Bi s. The lack of contribution of Bi p to the latter levels is consistent with the regular bioctahedral coordination and lack of a stereochemically active lone pair.

**Figure 10:**
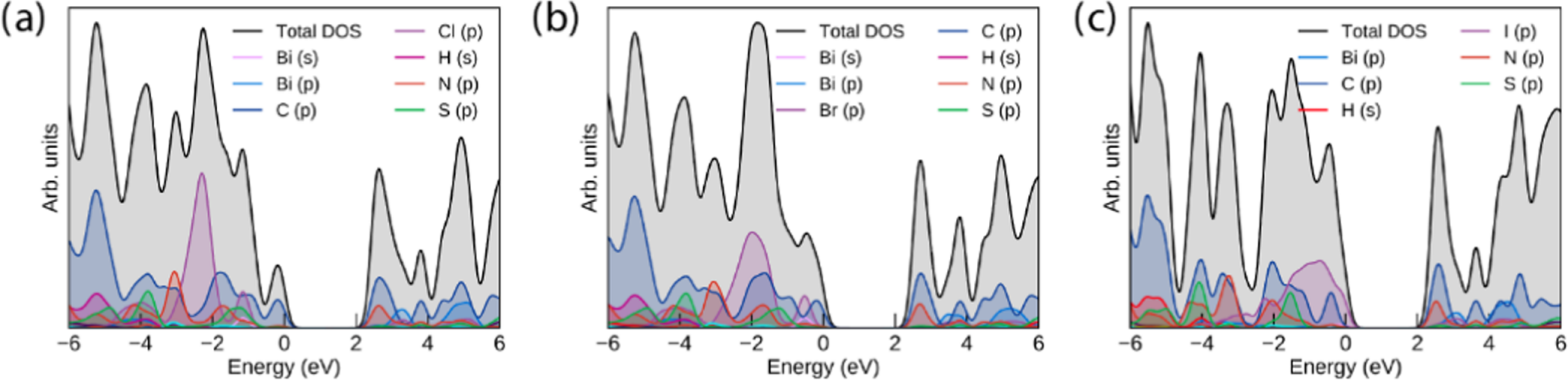
HSE06+SOC densities of states (DOS) of compounds **1–3 (a–c)**, plotted using the sumo package.^65^ All densities of states have *E* = 0 eV set to the valence band maximum; a Gaussian smearing of 0.2 eV is applied to all states.

As bismuth is a heavy element and additionally iodine in compound **3**, it is necessary to also explore the effect of spin–orbit coupling on the DOS of the three compounds: the HSE06+SOC densities of states are included in Figure 10. While SOC has an appreciable effect on the Bi p states in the conduction band of all three compounds, splitting them by 1 eV, this is still insufficient for any to lie lower than the unoccupied states of the organic ligand, retaining the ^1^LC character of the fundamental gap in compounds **1** and **2**. In compound **3**, the additional splitting of the iodine p states in the valence band is small but sufficient to lead to their further dominating the valence band maximum.

With the HSE06+SOC results, we can attempt to quantitatively associate the band-to-band transitions with our experimental findings. The fundamental band gaps of the chloride and bromide are very similar due to both being the aforementioned ^1^LC, with the former being 2.64 eV. This appears to underestimate the 392 nm transition maximum seen experimentally; however, while there is minimal dispersion in the band structure, there is a series of bands nearby in energy to both VBM and CBM, covering a 0.2 eV range in each case—as such, with the fundamental transition at 470 nm lying lie near the absorption onset, the majority of this set of transitions will occur at around 3 eV. This is in excellent alignment with the experiment, even with the absence of explicit electron–phonon coupling within the theoretical results, and further supports the assignment of this transition.

The theoretical results are then also in agreement with the absorption characteristics of the iodide: with the iodide states acting to dominate the valence band and a lowered set of Bi p states in the conduction band, the fundamental transition is expected to be from iodide states to organic but with a LMCT transition close in energy (and with potential MC character due to the contribution of Bi s near the VBM). The lowered band gap of 2.49 eV in compound **3** is also in good agreement with the onset at ∼500 nm of the concentration-dependent absorption, as shown in Figure 4b. Overall, the periodic DFT calculations demonstrate a correspondence with the solution-phase absorption, and thus, similar photophysical behavior may be possible in the solid state.

#### Discussion

The detailed absorption and photoluminescence studies of **1**, **2**, and **3** show the effect of the solvent and the ancillary ligand over the photophysical properties of the bismuth bioctahedral complexes. The comparison of ground-state absorption properties of the ligand with the complexes shows that the absorption spectra of the complexes are strongly dependent on the choice of the ancillary ligand bound to the metal center. As the size of the halogen bound to the bismuth center increases, the electron-donation ability of the halogen increases, which significantly affects the ground-state absorption of the complexes, and the results are further supported by the theoretical calculations. Additionally, the change in the ancillary ligand from chlorine, bromine, to iodine causes the halides to dominate the ground-state absorption of the complexes and reduces the contribution of the organic ligand in affecting the ground-state properties, which is also in agreement with theoretical studies. This behavior is further confirmed by the observation of a lower-energy absorption band (440–525 nm) in the case of complexes **3** and **4** in DMF, while no such band is observed in the case of other complexes. Furthermore, the comparison of the absorption spectra recorded in ACN for **L**, **3**, and **4** at a concentration of 10 μM also proves the contribution of the inorganic part to the ground-state properties of the complexes.

**Figure 11:**
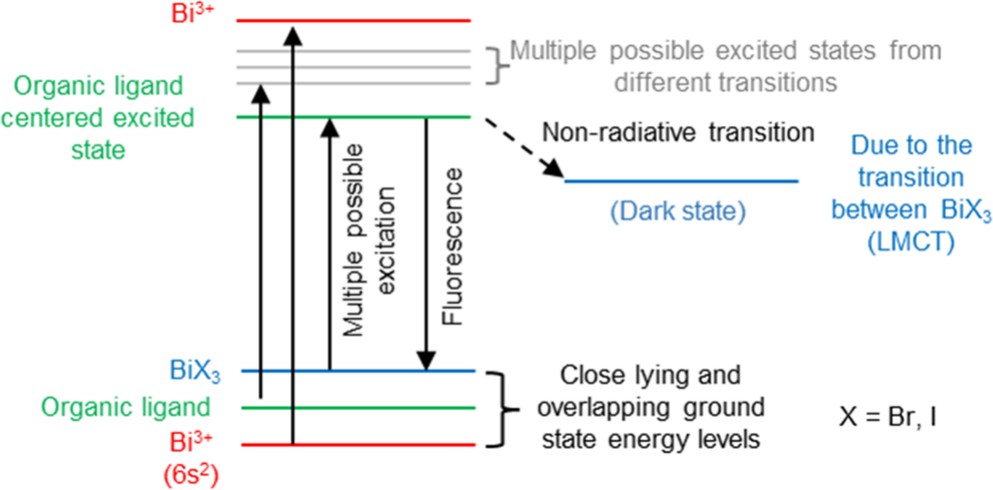
Qualitative energy level diagram showing the alignment of different energy levels for complexes **2** and **3** in solutions.

The photoluminescence analysis and comparison of a series of complexes with ligand **L** show that the lower-energy emissive excited state is the organic ligand centered in all of the solvents (Figure 11). However, the change in the ancillary ligand causes the lifetime of the emissive singlet state to vary depending on the solvent used. The observation of the lowest singlet state lifetime for all of the complexes in ACN suggests that the excited-state phenomenon, which otherwise is absent in other solvents, controls the dynamics in the ACN. Moreover, the photoluminescence quantum yield measurements also suggest that all of the complexes are poorly emissive in ACN, and these results are further supported by titration studies between DMF and ACN. As explained earlier, the titration of the ligand with an increasing amount of the salts in ACN causes a significant reduction in emission intensity in all cases. Comparing these excited-state results with the ground-state properties in ACN for **3** suggests that ACN strongly influences the ground- and excited-state properties of the complexes. Taking the idea from the previous literature published on the photochemistry of the Bi(III)I_6_^3–^ ions^60,64^ and halide photochemistry, we assume that the lower-energy absorption band (450–550 nm in DMF and 440–525 nm in ACN) is purely the halide LMCT in nature. Additionally, when the complexes were excited at 440–525 nm in ACN, no emission was observed, which further confirms that these bands lead to the population of LMCT states, which are generally known to be nonemissive. Moreover, the reduction in the intensity and quenching of the ligand excited state with an increasing amount of bismuth salt during the titration can also be explained by the presence of a lower-energy emissive state (Figure 11) in the case of **2-Br** and **3-I**. We hypothesize that the quenching of the **L** excited state is caused by the presence of the lower-energy halide-based LMCT state, which depopulates the excited state, as shown qualitatively in the energy diagram in Figure 11. We suggest that this is the intrinsic property of the bismuth halide-based bioctahedral complexes and hence makes these complexes less suitable for high-emission-based applications like OLEDs. Moreover, comparing the initial transient absorption studies of the complexes in DMF with the Bi(III)I_6_^3–^ ions reported in the literature, the results show that only one triplet excited-state species is present in the complexes in DMF. The observation of the single species, especially in **2** and **3**, is in contrast to the reported studies, which show that halobismuthate(III) complexes undergo photodissociation and excited-state reactions to give rise to long-lived multiple species.^60,62,63^ These initial studies further confirm the stability of the complexes in the excited state and thus provide the opportunity to explore the excited-state dynamics of the bismuth-based coordination complexes for optoelectronics applications.

## Conclusions

In summary, we synthesized and investigated the photophysical properties of the series of bismuth–halide-based coordination complexes. The results demonstrate that both the conventional and solvothermal synthesis can be utilized to synthesize the bismuth-based bioctahedral complexes; however, conventional synthetic conditions provide more control over the reaction products, hence leading to the formation of a single-phase product. Also, the kinetic NMR studies provide the stability of complexes in the solution and show that the complexes are stable in solution to study their fundamental properties. The detailed photophysical studies of the complexes in the solution phase show that ground- and excited-state properties are solvent-dependent and strongly controlled by the inorganic part of the complexes. The choice of bismuth halide excessively affects the absorption coefficient as well as the excited-state photophysical properties of the complexes. The presence of bismuth iodide in the coordination complexes extends the absorption spectra to the visible region and also causes the quenching of the organic ligand-based excited state due to the lower-energy BiI_3_-based LMCT state. Moreover, the computational studies further support the experimental data and show minimum hybridization between the electronic states of the inorganic and organic parts. Finally, the combined experimental and theoretical studies provide the opportunity to understand the ground- and excited-state electronic behavior of the bismuth halide-based coordination complexes and show that the presence of the electronically rich donor part in the organic ligand does not mix the electronic states of the inorganic and organic parts. Hence, we hypothesize that the future development of such coordination complexes should be performed with the electronically deficient organic ligands in order to increase the hybridization of the organic and inorganic parts.

## Supporting Information Available

Detailed synthetic procedures, X-ray analysis, UV–vis spectroscopy, ultraviolet photoelectron spectroscopy, steady-state emission spectroscopy, time-correlated single photon counting, and transient absorption spectroscopy analyses (PDF)

## Accession Codes

CCDC 2221714–2221716 and 2293384 contain the supplementary crystallographic data for this paper. These data can be obtained free of charge via www.ccdc.cam.ac.uk/data_request/cif, or by emailing data_request@ccdc.cam.ac.uk, or by contacting The Cambridge Crystallographic Data Centre, 12 Union Road, Cambridge CB2 1EZ, U.K.; fax: +44 1223 336033.
