# Supplementary material for: Exploring Bismuth Coordination Complexes as Visible-Light Absorbers: Synthesis, Characterization, and Photophysical Properties
Source: Inorg Chem. 2023 Dec 15;63(1):416–30. doi: 10.1021/acs.inorgchem.3c03290 (PMC10777407; doi:10.1021/acs.inorgchem.3c03290)
Supplement: Supplementary file 1 — ic3c03290_si_001.pdf [file ic3c03290_si_001.pdf]

Supporting Information

Exploring Bismuth coordination complexes as visible light absorbers: Synthesis, characterization and photophysical properties

Harsh Bhatia[a,*], Junjun Guo[a], Christopher N. Savory[a,b], Martyn Rush[c], David Ian James[d], Avishek Dey[a], Charles Chen[a], Dejan-Krešimir Bučar[a], Tracey M. Clarke[a], David O. Scanlon[a,b,e], Robert G. Palgrave[a], Bob C. Schroeder [a*]

[a] Department of Chemistry, University College London, 20 Gordon Street, London WC1H 0AJ, United Kingdom.

[b] Thomas Young Centre, University College London, London WC1E 6BT, United Kingdom.

[c] Polysolar Ltd, High Cross, Aurora Cambridge at BAS, Madingley Rd, Cambridge, CB3 0ET, United Kingdom.

[d] Johnson Matthey Technology Centre, Blount's Court, Sonning Common, Reading, RG4 9NH, United Kingdom.

[e] Diamond Light Source Ltd., Diamond House, Harwell Science and Innovation Campus, Didcot, Oxfordshire OX11 0DE, United Kingdom.

[*]Email: h.bhatia@ucl.ac.uk, b.c.schroeder@ucl.ac.uk

Table of Contents

1. Synthetic Schemes 2
2. NMR spectra 4
3. Single crystal x-ray diffraction data 10
4. Powder x-ray diffraction data 16
5. Spectroscopic data 17
6. Density functional theory 27
7. Ultraviolet photoelectron spectroscopy 28
8. References 29

1. Synthetic Schemes

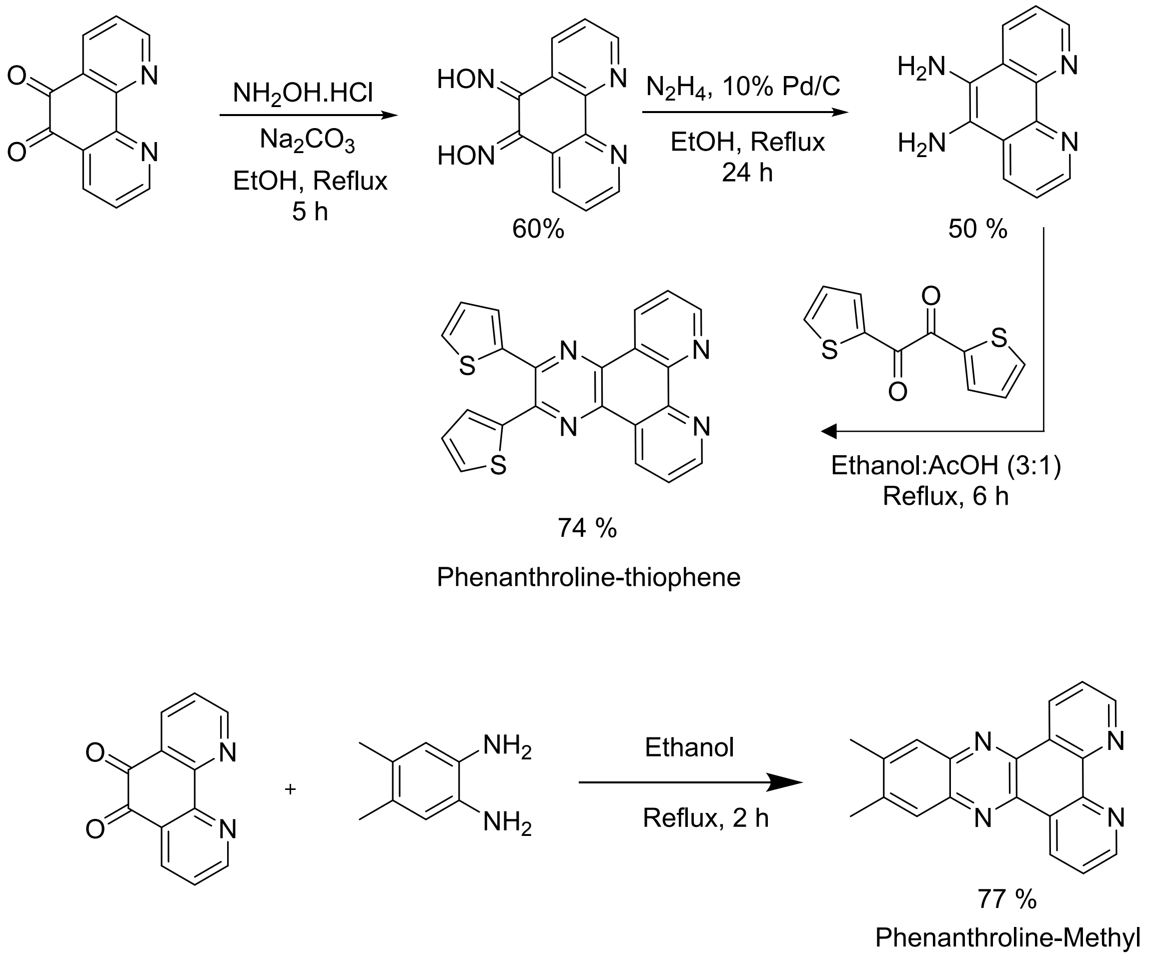

Scheme S1. Synthetic scheme for ligand phenanthroline-thiophene and phenanthroline-methyl.

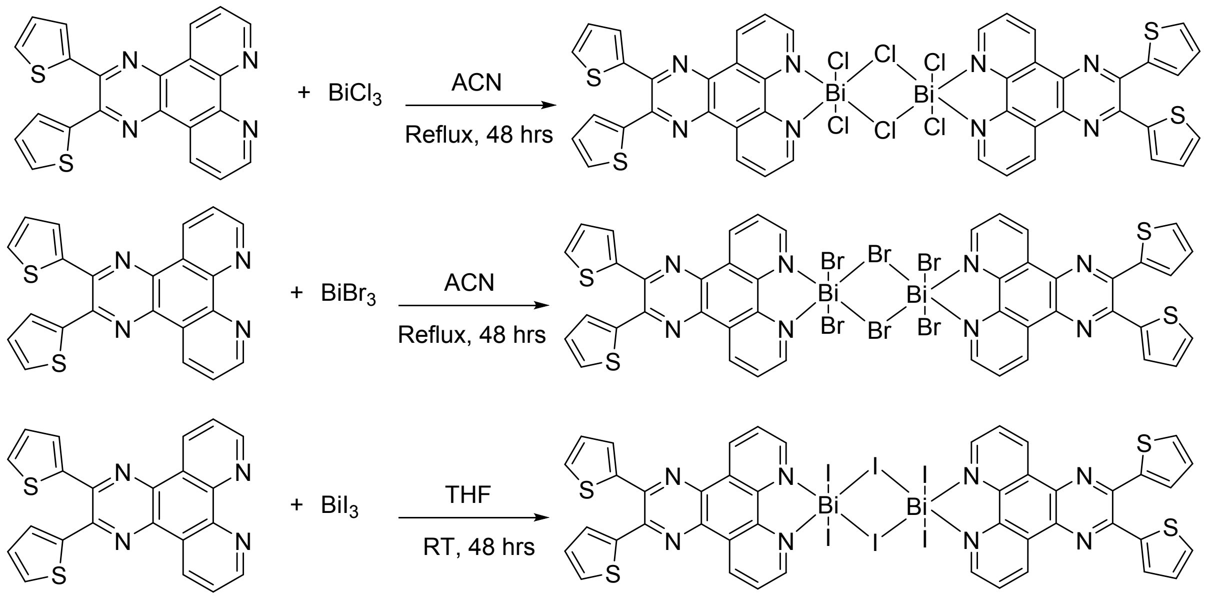

Scheme S2. Synthetic scheme for conventional synthesis of $Bi_2Cl_6(L)_2$ (**1**), $Bi_2Br_6(L)_2$ (**2**) and $Bi_2I_6(L)_2$ (**3**).

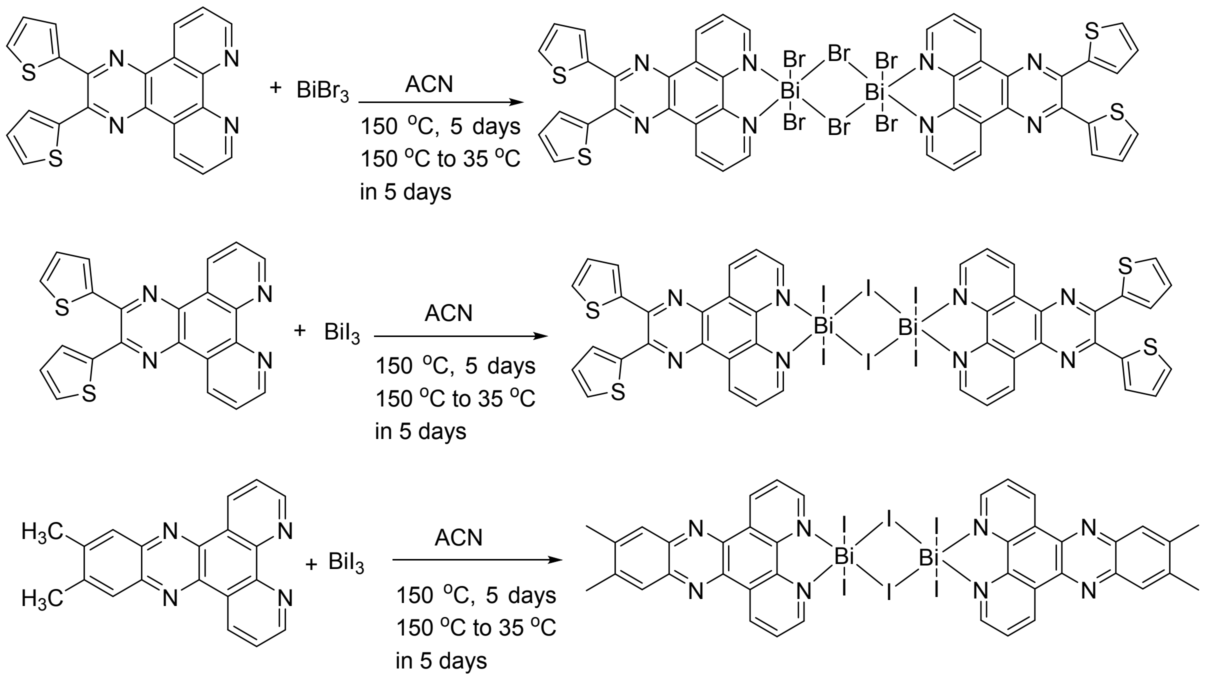

Scheme S3. Synthetic scheme for solvothermal synthesis of $Bi_2Br_6(L)_2$ (**2**), $Bi_2I_6(L)_2$ (**3**) and $Bi_2I_6(L')_2$ (**4**).

2. NMR spectra

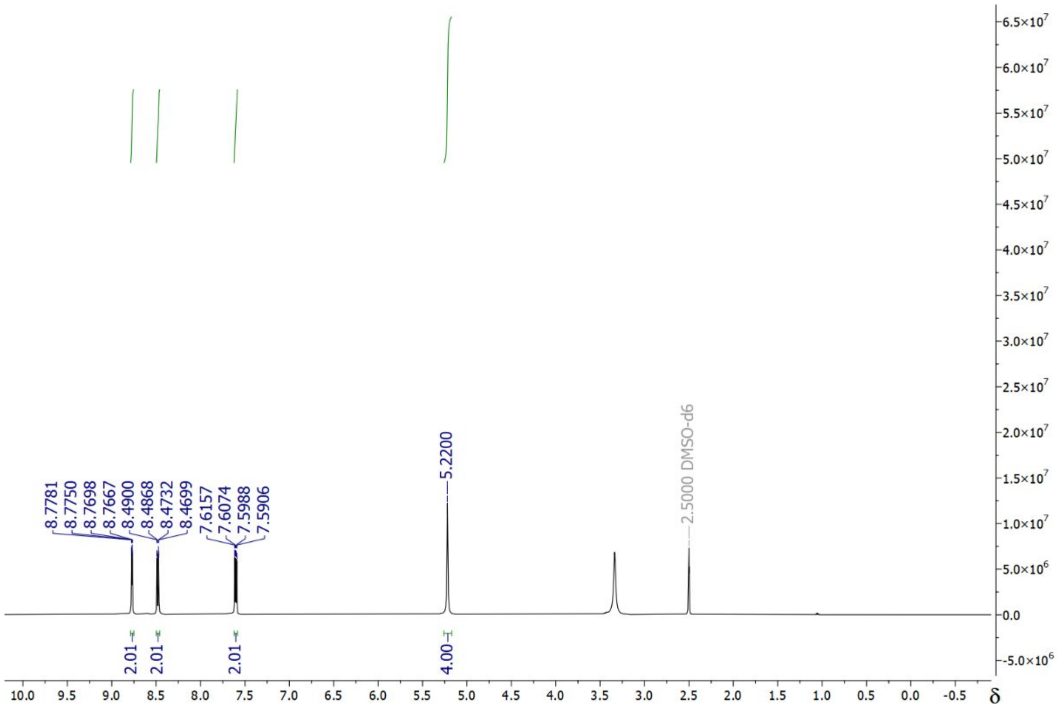

Figure S1. The ^{1}H NMR (500 MHz in DMSO-d_6, RT) of 5,6-diamino-1,10-phenanthroline.

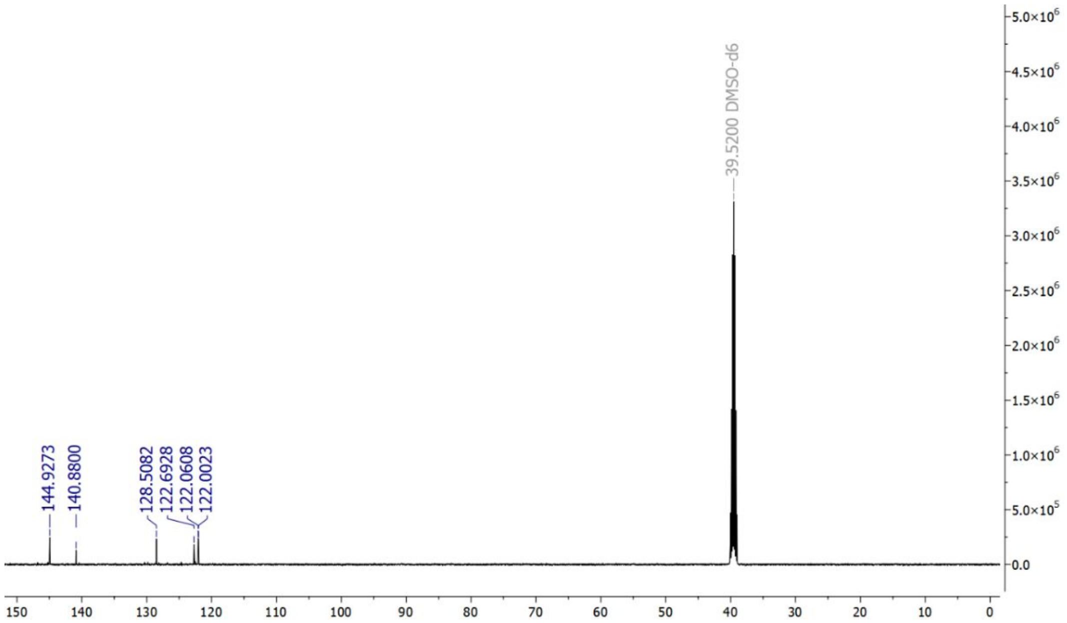

Figure S2. The ^{13}C NMR (125 MHz in DMSO-d_6, RT) of 5,6-diamino-1,10-phenanthroline.

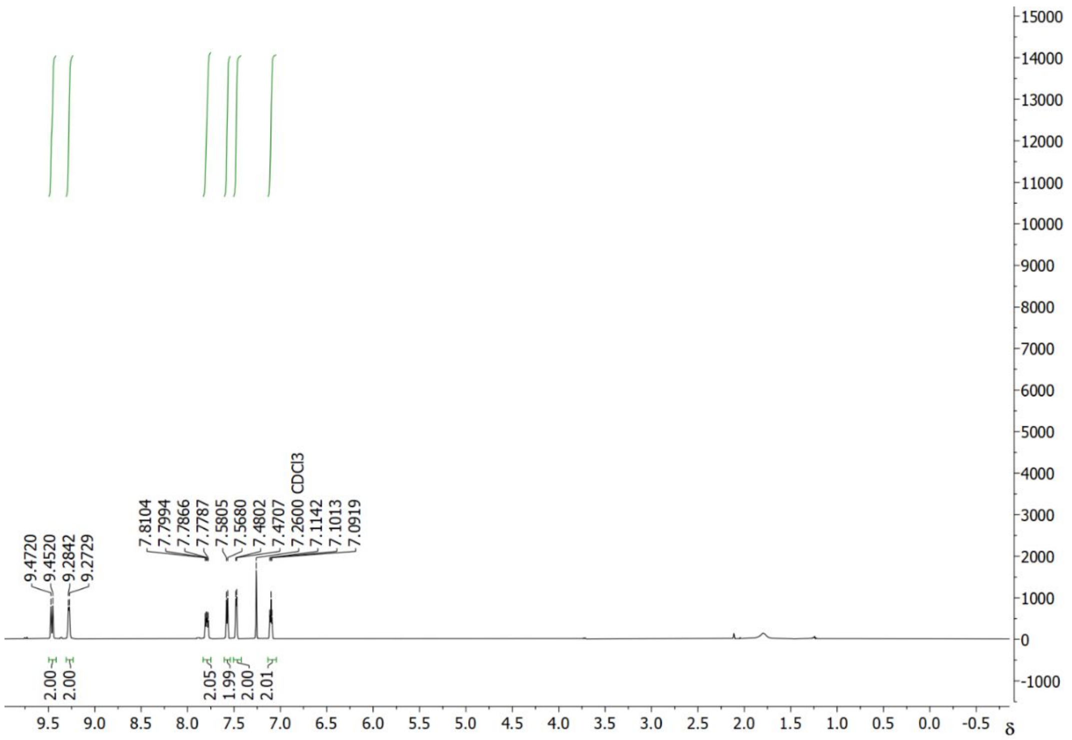

Figure S3. The ^{1}H NMR (500 MHz in $CDCl_3$, RT) of 2,3-di(thiophen-2-yl)pyrazino[2,3-*f*][1,10]phenanthroline (**L**).

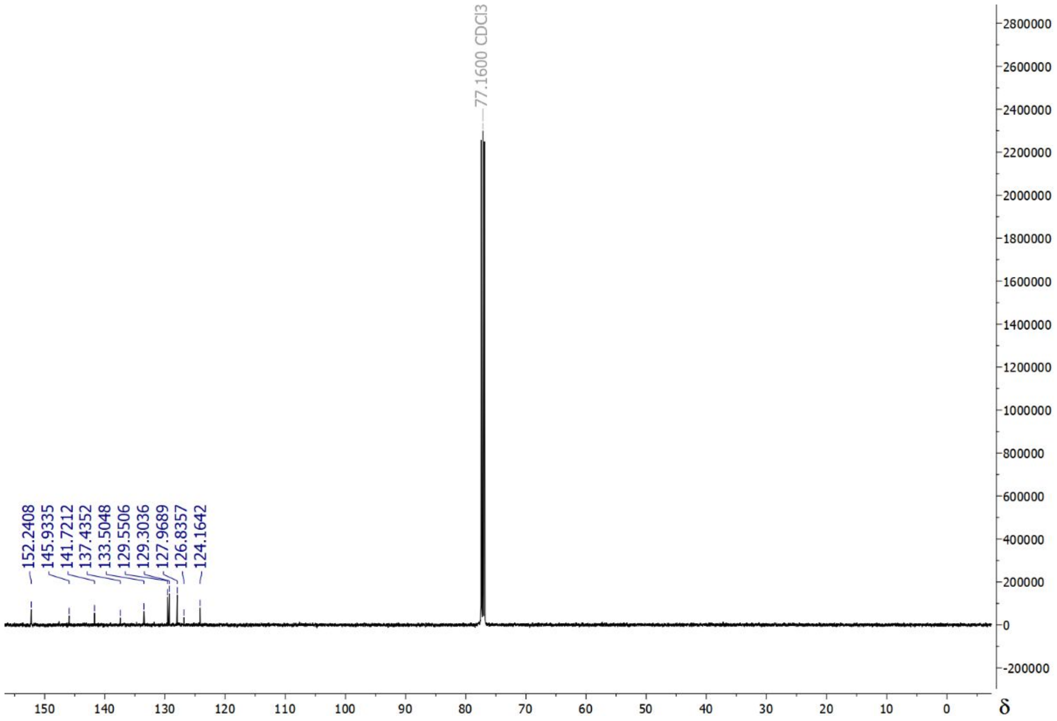

Figure S4. The ^{13}C NMR (125 MHz in $CDCl_3$, RT) of 2,3-di(thiophen-2-yl)pyrazino[2,3-*f*][1,10]phenanthroline (**L**).

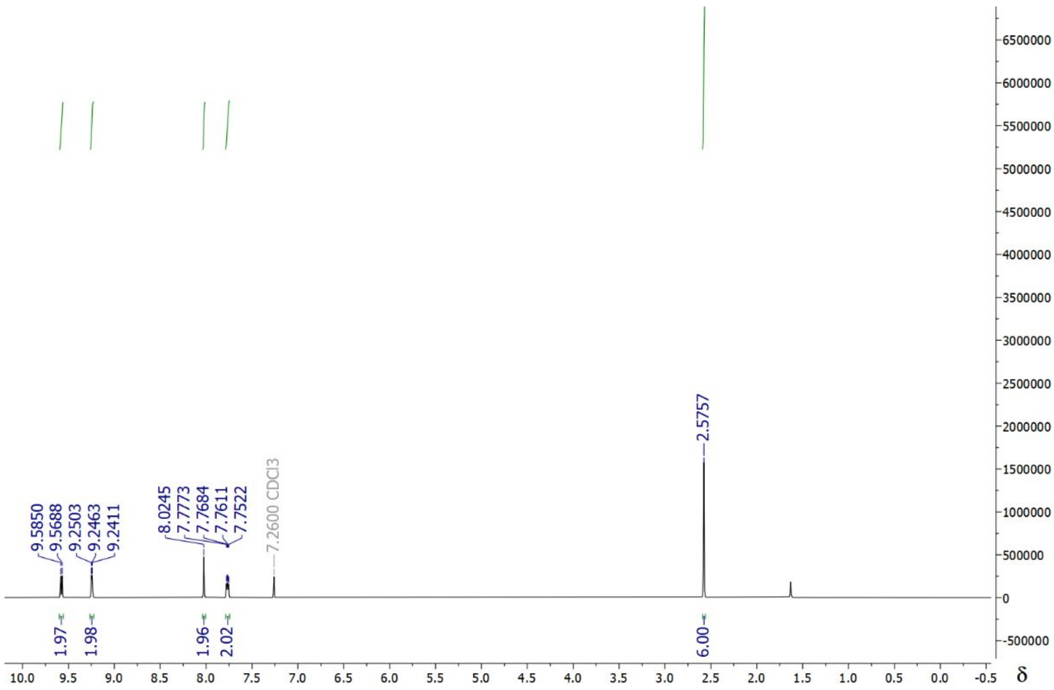

Figure S5. The ^{1}H NMR (500 MHz in $CDCl_3$, RT) of 11,12-dimethyldipyrido[3,2-*a*:2',3'-*c*]phenazine (**L′**).

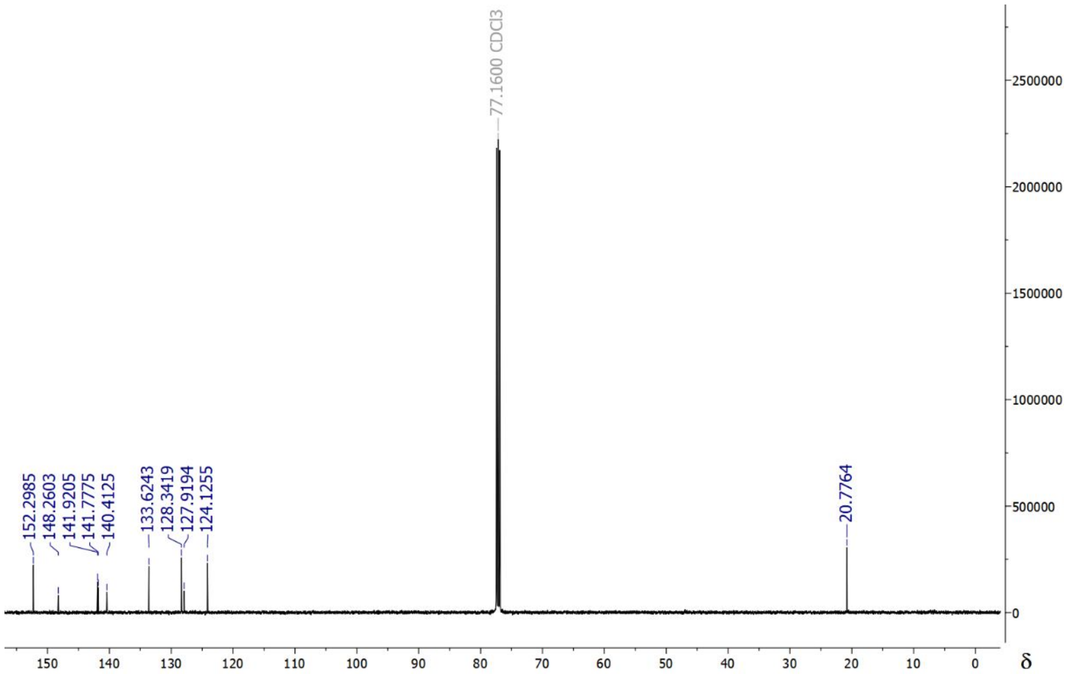

Figure S6. The ^{13}C NMR (125 MHz in $CDCl_3$, RT) of 11,12-dimethyldipyrido[3,2-a:2',3'-c]phenazine (**L′**).

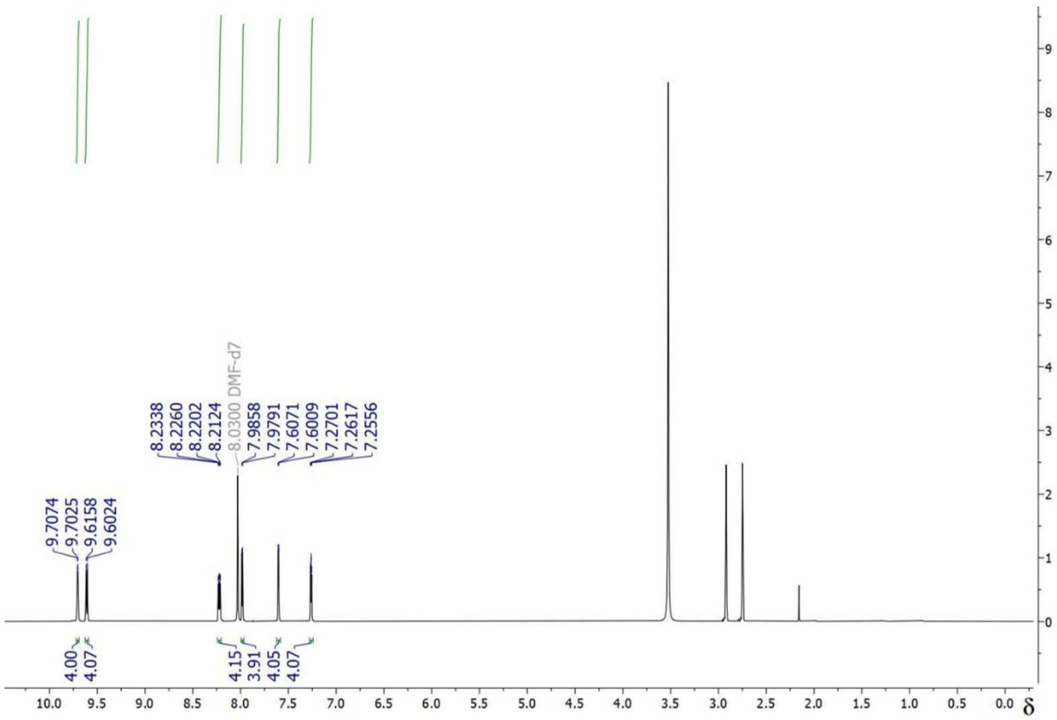

Figure S7. The ^{1}H NMR (600 MHz in DMF-d_7, RT) of complex **1**.

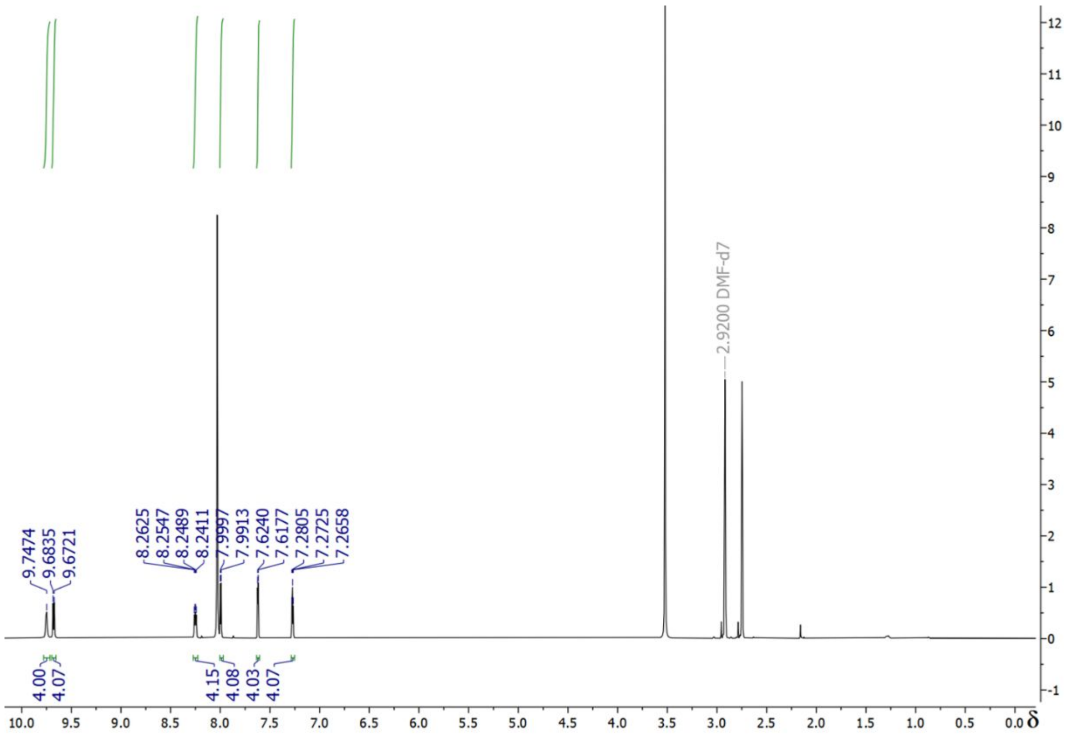

Figure S8. The ^{1}H NMR (600 MHz in DMF-d_7, RT) of complex **2**.

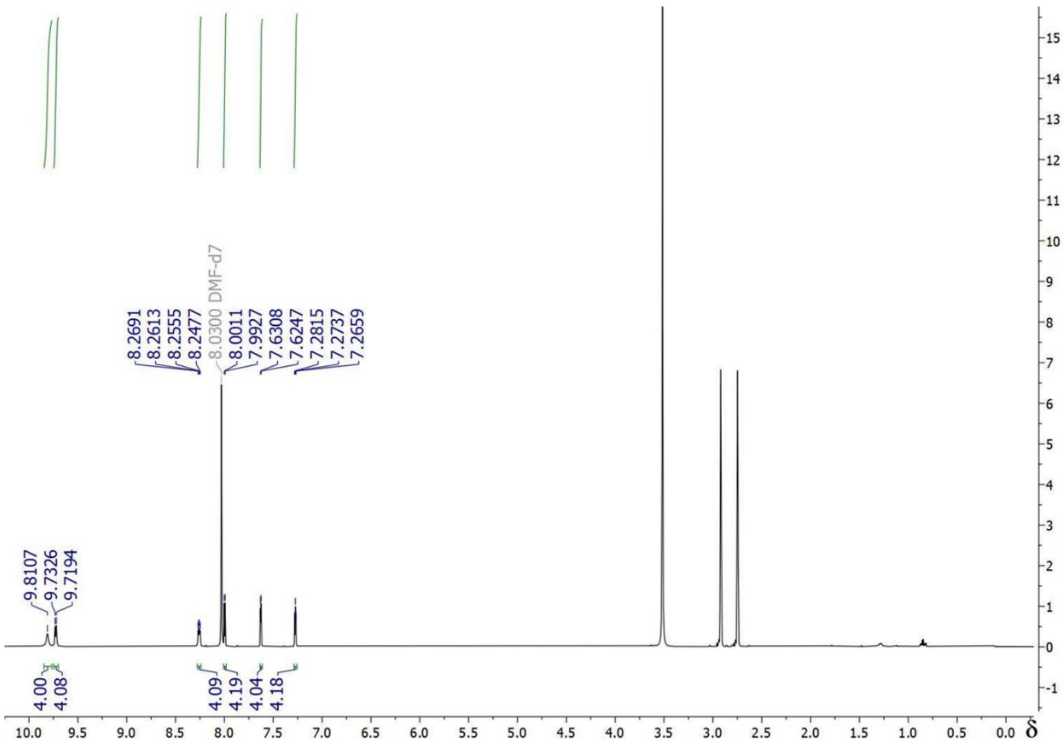

Figure S9. The ^{1}H NMR (600 MHz in DMF-d_7, RT) of complex **3**.

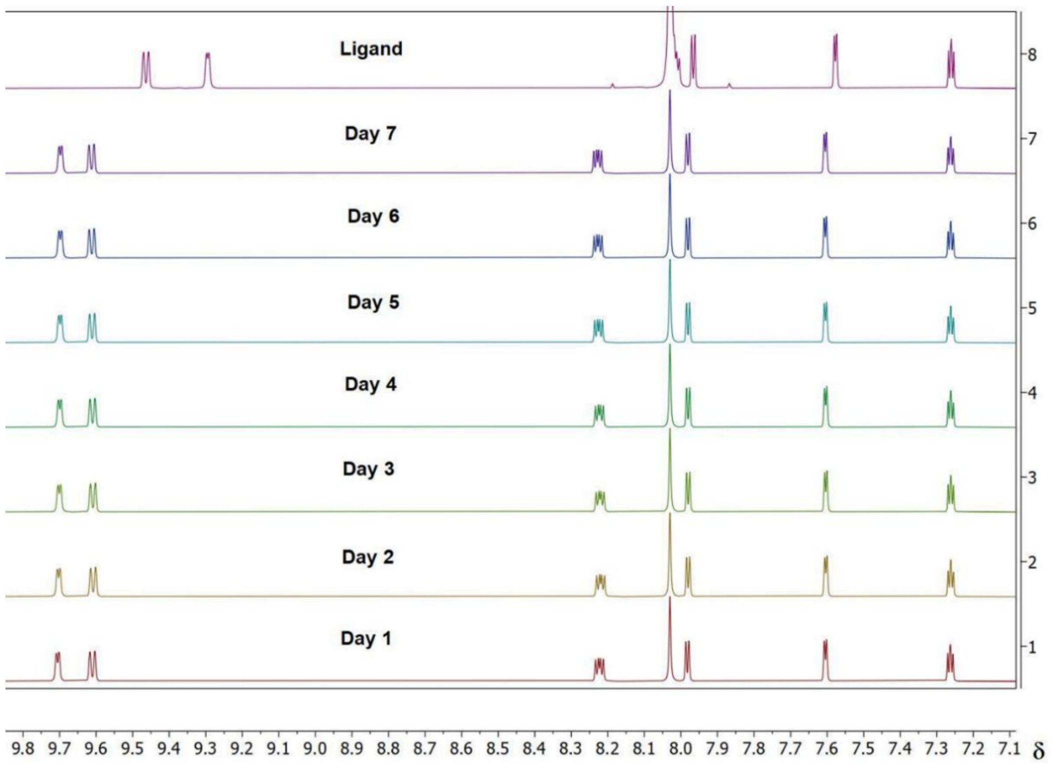

Figure S10. The ^{1}H NMR (600 MHz in DMF-d_7, RT) kinetics of complex **1**.

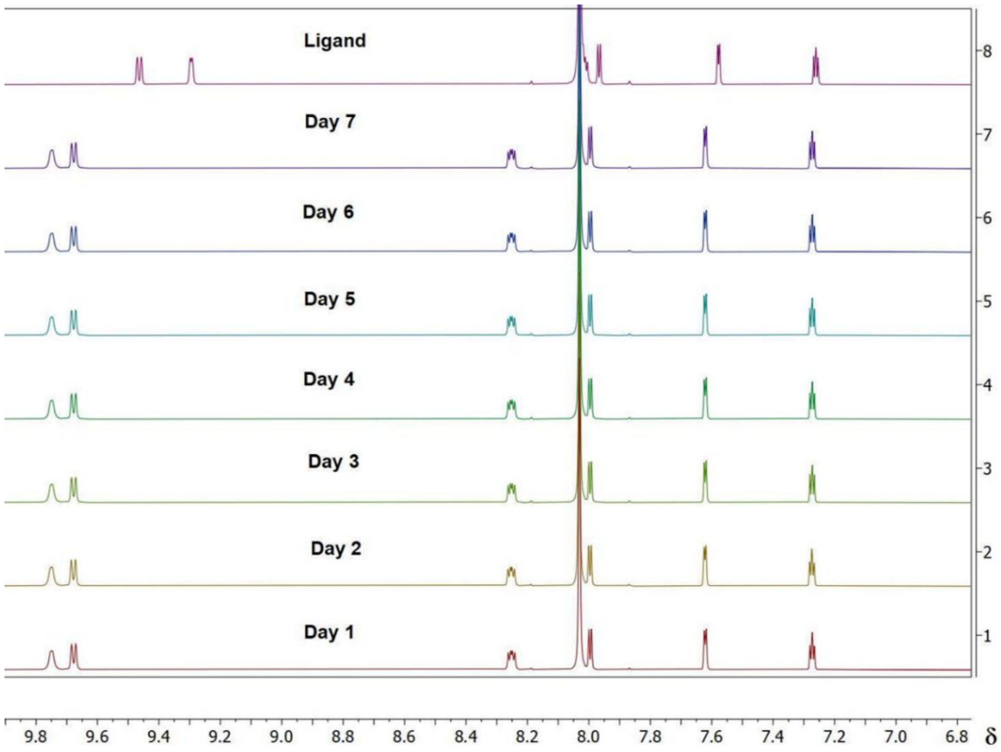

Figure S11. The ^{1}H NMR (600 MHz in DMF-d_7, RT) kinetics of complex **2**.

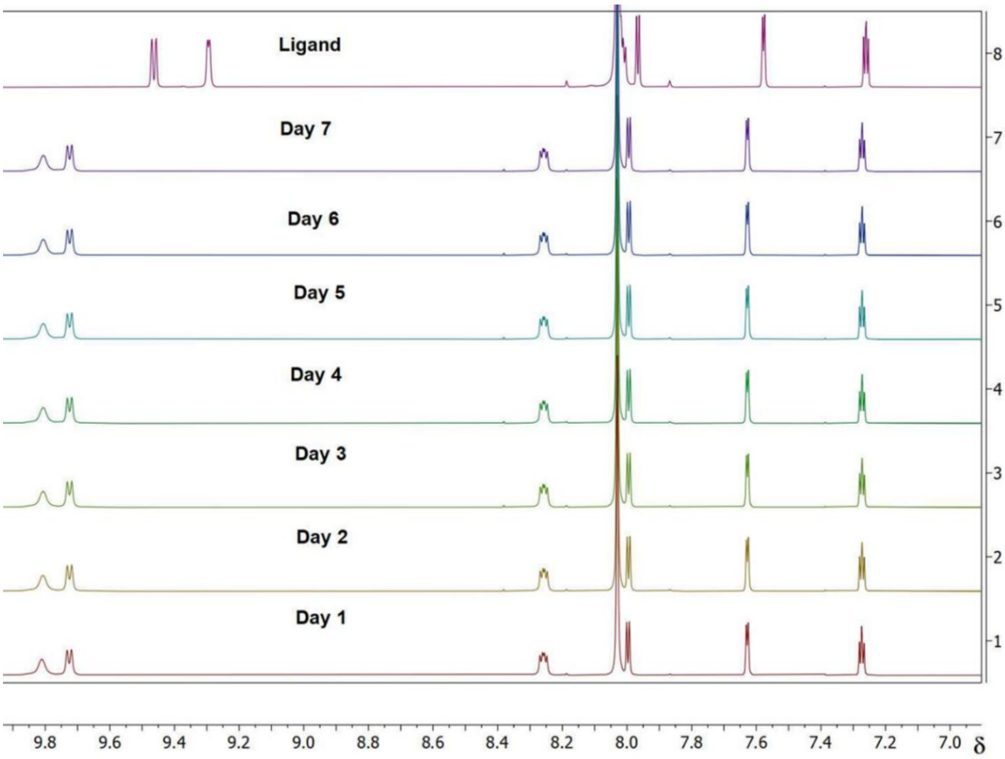

Figure S12. The ^{1}H NMR (600 MHz in DMF-d_7, RT) kinetics of complex **3**.

3. Single crystal x-ray diffraction data

The diffraction data for complexes **1-4** were collected on a four-circle *Agilent SuperNova* (Dual Source) single crystal X-ray diffractometer using a micro-focus CuK_α X-ray beam (λ = 1.54184 Å) and an *Atlas* CCD plate detector (in case of **1** and **2**) or a *HyPix-Arc 100* hybrid pixel array detector (in case of **3** and **4**). The sample temperatures were controlled with an *Oxford Instruments* cryojet. All data were processed using the *CrysAlis^{Pro}* programme package from *Rigaku Oxford Diffraction*.[1] The crystal structures were solved with the *SHELXT* programme,[2] used within the *Olex2* software suite,[3] and refined by least squares on the basis of F^2 with the *SHELXL*[4] programme using the *ShelXle* graphical user interface.[5] All non-hydrogen atoms were refined anisotropically by the full-matrix least-squares method. Hydrogen atoms associated with carbon atoms were refined isotropically [U_{iso}(H) = 1.2U_{eq}(C)] in geometrically constrained positions. The F_o–F_c difference maps were in all cases used to identify disordered thiophen moieties. These disorders were modelled using the SAME similarity restraint command in *SHELXL*.[4] The anisotropic parameters of the disordered thiophen groups were restrained or constrained using the SIMU or EADP commands in *SHELXL*.[4] The crystallographic and refinement parameters of **1−4** are given in **Table S1**. The asymmetric units of the crystal structures of **1−4** are shown in **Figures S13 – S16**.

Table S1. Crystallographic and refinement parameters of **1−4.**

	1	2	3	4
empirical formula	$C_{44}H_{24}Bi_2Cl_6N_8S_4$	$C_{44}H_{24}Bi_2Br_6N_8S_4$	$C_{44}H_{24}Bi_2I_6N_8S_4$	$C_{42}H_{31}Bi_2I_6N_9$
M_r / g mol^{-1}	1423.61	1690.37	1972.31	1841.12
crystal system	orthorhombic	orthorhombic	monoclinic	monoclinic
space group	*Pbca*	*Pbca*	*C*2/*c*	*P*2$_1$/*c*
a / Å	22.3168(2)	23.02977(14)	7.97000(10)	14.2660(2)
b / Å	7.52760(10)	7.64231(5)	34.3208(4)	21.9654(3)
c / Å	26.3628(2)	26.27273(16)	18.2437(2)	8.30160(10)
α / °	90	90	90	90
β / °	90	90	93.6230(10)	101.1810(10)
γ / °	90	90	90	90
V / Å^3	4428.74(8)	4624.02(5)	4980.35(10)	2552.00(6)
Z	4	4	4	2
ρ_{calc} / g cm^{-3}	2.135	2.428	2.630	2.396
T / K	150.0(1)	150.0(1)	150.0(1)	150.0(1)
μ / mm^{-1}	20.827	22.855	44.866	42.192
F(000)	2704	3136	3568	1660
crystal size / mm^3	0.30 × 0.08 × 0.04	0.24 × 0.10 × 0.02	0.28 × 0.07 × 0.03	0.21 × 0.11 × 0.04
radiation	CuK_α (λ = 1.54184 Å)	CuK_α (λ = 1.54184 Å)	CuK_α (λ = 1.54184 Å)	CuK_α (λ = 1.54184 Å)
2ϑ range for data collection / °	7.790 – 133.198	6.728–133.194	7.078 – 133.200	7.366 – 133.166
index ranges	$-26 \le h \le 26$<br>$-8 \le k \le 8$<br>$-31 \le l \le 31$	$-27 \le h \le 27$<br>$-8 \le k \le 9$<br>$-31 \le l \le 31$	$-7 \le h \le 9$<br>$-40 \le k \le 40$<br>$-21 \le l \le 19$	$-16 \le h \le 16$<br>$-26 \le k \le 26$<br>$-9 \le l \le 9$
number of collected reflections	73726	109531	16498	54719
unique reflections	3896	4081	4404	4484
number of unique reflections	3758 [$I > 2\sigma(I)$]	3977	4247	4218
R_{int}	0.0360	0.0422	0.0327	0.0490
R(*F*), *F* > 2σ(F)	0.0155	0.0154	0.0325	0.0269

$wR(F^2)$, $F > 2\sigma$(F)	0.0165	0.0160	0.0334	0.0290
$R(F)$, all data	0.0383	0.0375	0.0869	0.0732
$wR(F^2)$, all data	0.0388	0.0378	0.0877	0.0745
Δ_r (max., min.) e Å^{-3}	0.494/−0.693	0.388/−0.503	2.200/−1.573	2.101/−1.284
CCDC deposition number	2221714	2221715	2221716	2293384

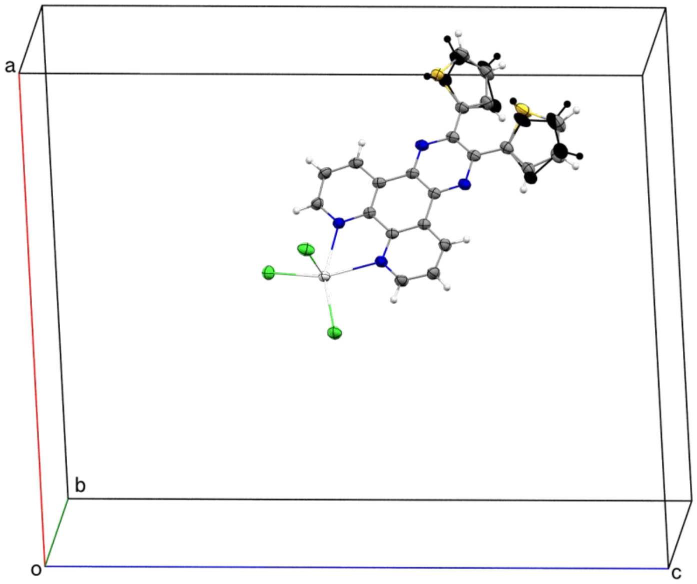

Figure S13. The asymmetric unit of compound **1**. The thermal ellipsoids are drawn at the 50% probability level. Colour scheme: carbon – dark grey, hydrogen – white, bismuth – light grey, chlorine – green, nitrogen – blue, sulfur – yellow. The minor occupancy sites are highlighted in black.

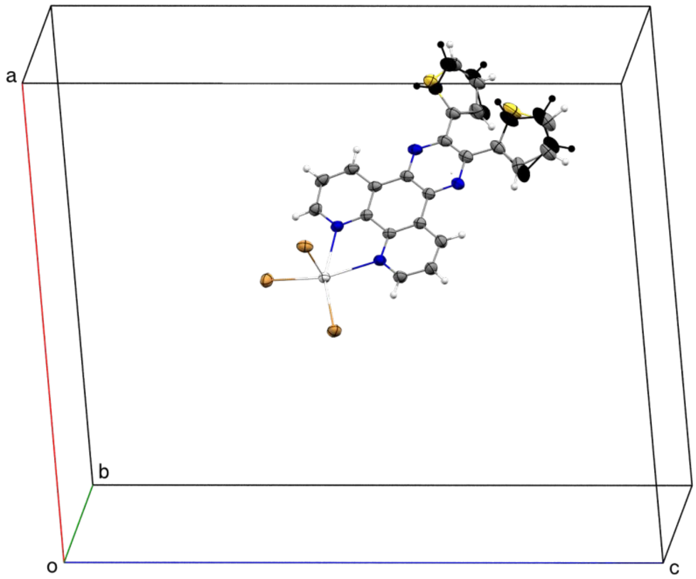

Figure S14. The asymmetric unit of compound **2**. The thermal ellipsoids are drawn at the 50% probability level. Colour scheme: carbon – dark grey, hydrogen – white, bismuth – light grey, bromine – brown, nitrogen – blue, sulfur – yellow. The minor occupancy sites are highlighted in black.

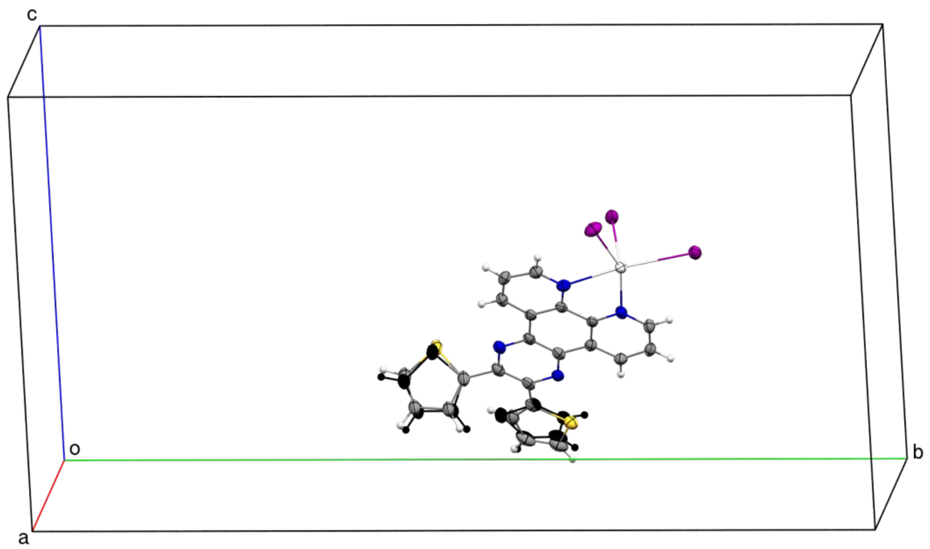

Figure S15. The asymmetric unit of compound **3**. The thermal ellipsoids are drawn at the 50% probability level. Colour scheme: carbon – dark grey, hydrogen – white, bismuth – light grey, Iodine – purple, nitrogen – blue, sulfur – yellow. The minor occupancy sites are highlighted in black.

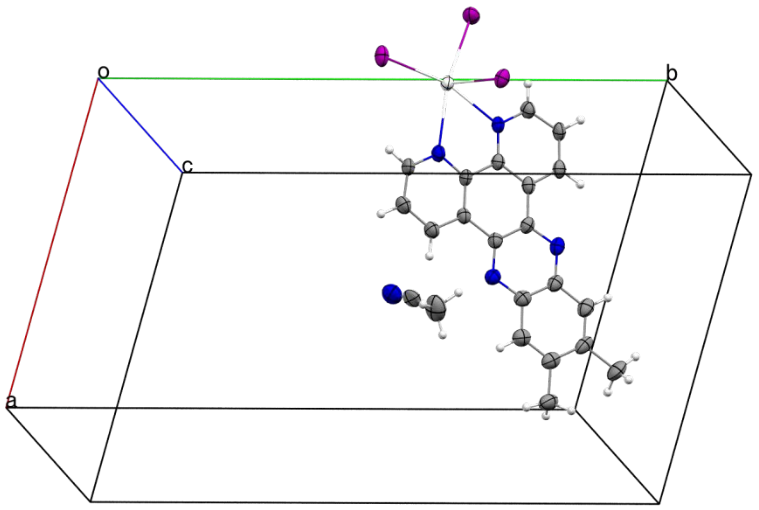

Figure S16. The asymmetric unit of compound **4**. The thermal ellipsoids are drawn at the 50% probability level. Colour scheme: carbon – dark grey, hydrogen – white, bismuth – light grey, Iodine – purple, nitrogen – blue.

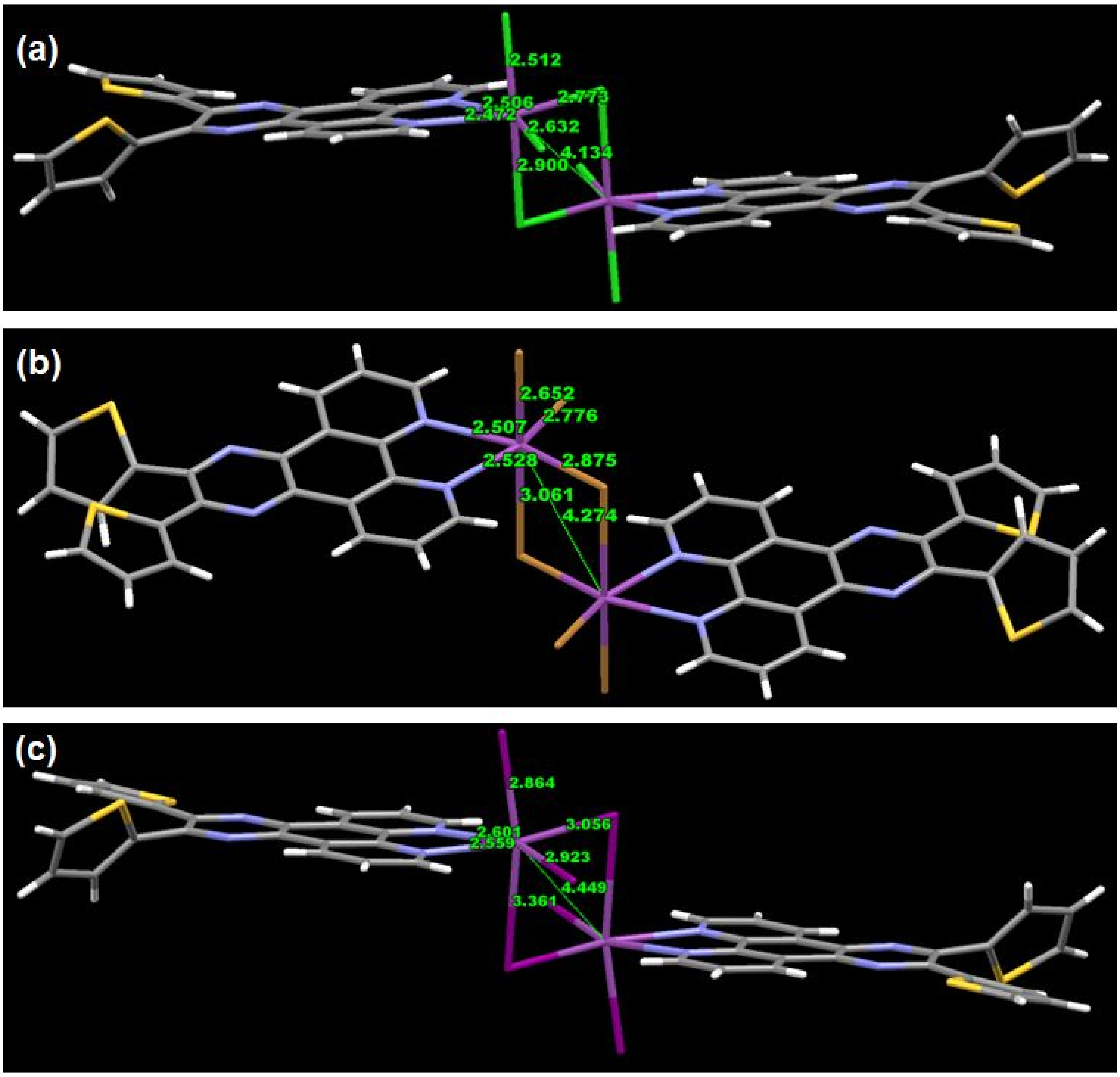

Figure S17. The SCXRD structures of (a) **1**, (b) **2**, and (c) **3** showing different bond lengths.

Table S2. Illustrating and comparing the bond distances in complexes **1**, **2**, and **3**.

**Complex**	**Bi–N$_L$** (Å)	**Bi–Bi** (Å)	**Bi–X** (X= Cl, Br, I)
**1**	2.472, 2.506	4.134	2.512, 2.632, 2.900, 2.773
**2**	2.507, 2.528	4.274	2.652, 2.776, 2.875, 3.061
**3**	2.559, 2.601	4.449	2.864, 2.923, 3.056, 3.361

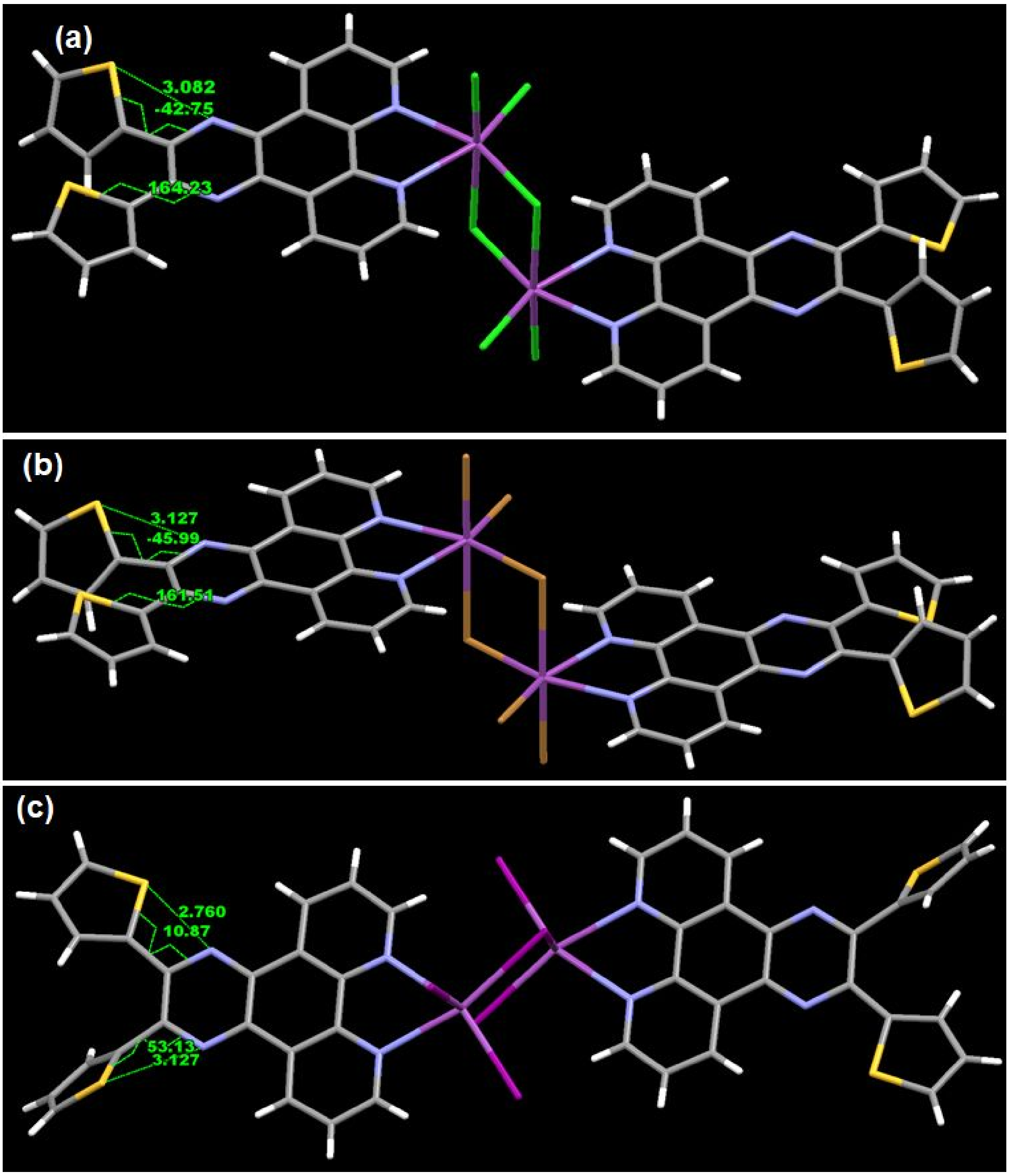

Figure S18. The dihedral angle between pyrazine and thiophene rings and the S•••N interactions of (a) **1**, (b) **2**, and (c) **3**.

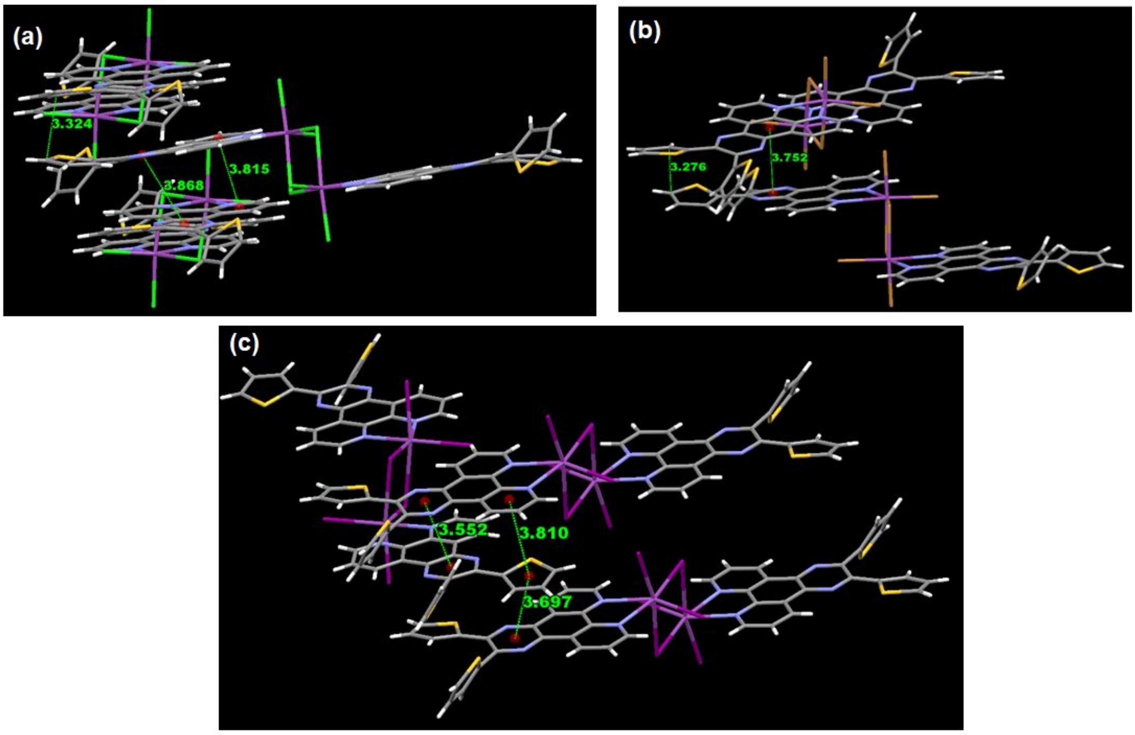

Figure S19. Intermolecular π-π interactions between the organic ligand of complexes (a) **1**, (b) **2**, and (c) **3**.

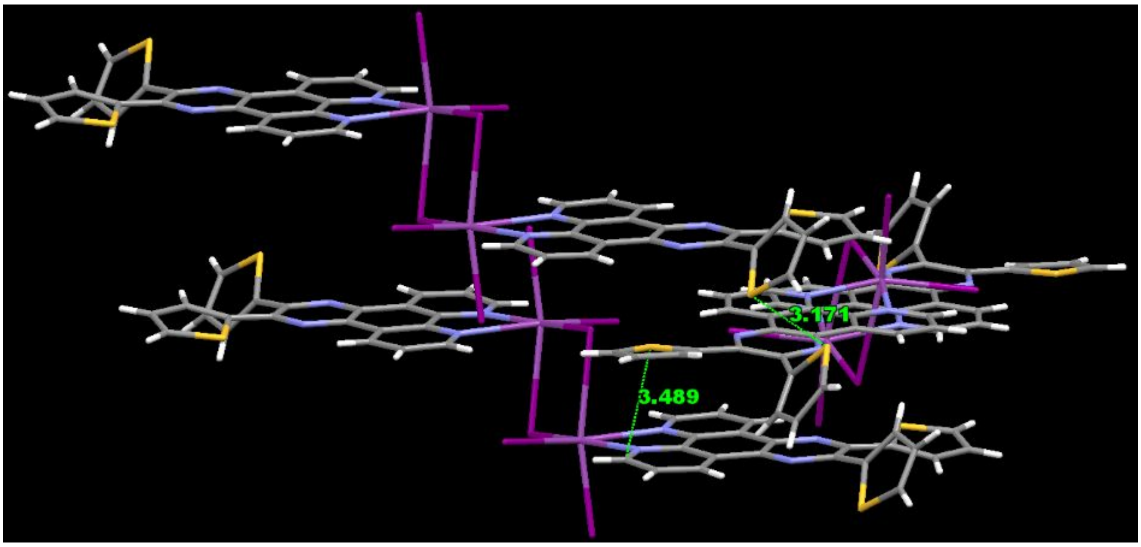

Figure S20. Intermolecular n-π and lp(S)●●●lp(S) interactions in **3**.

4. Powder x-ray diffraction data

Room temperature PXRD data sets were obtained using a STOE Stadi-P X-ray diffractometer equipped with a Cu tube set to 50 kV and 30 mA ($K_{\alpha 1}$ with $\lambda = 1.5406$ Å), a primary beam monochromator and Dectris Mythen detector. Measurements were made in thin-foil transmission mode with a 0.5° detector step in the 2θ range of 2° to 50°, and a counting time of 5 s per step. The data was acquired and treated using the STOE WinX^{POW} program (version 3.20).

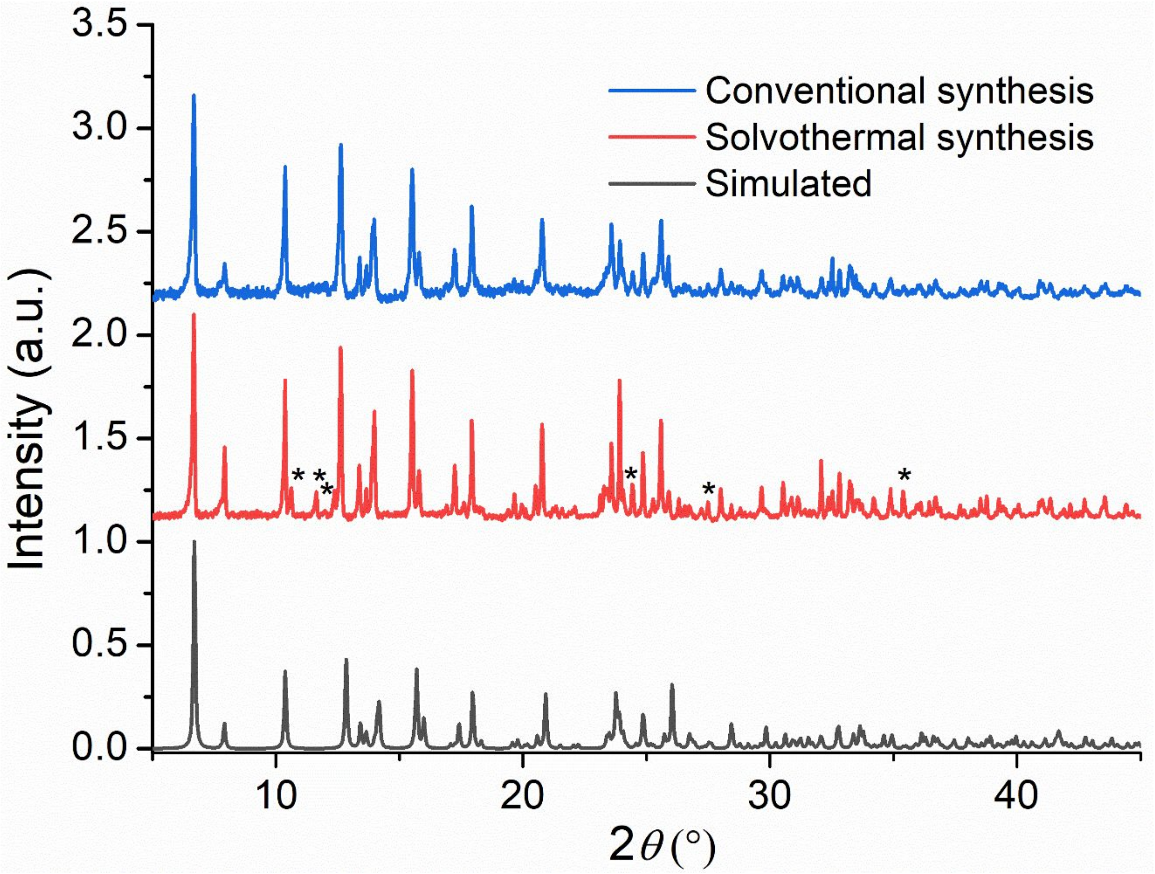

Figure S21. The comparison of experimentally collected and simulated PXRD patterns of complex **1**. The marked diffractions peaks (*) correspond to impurities in form of reagents and unidentified byproducts in **1**.

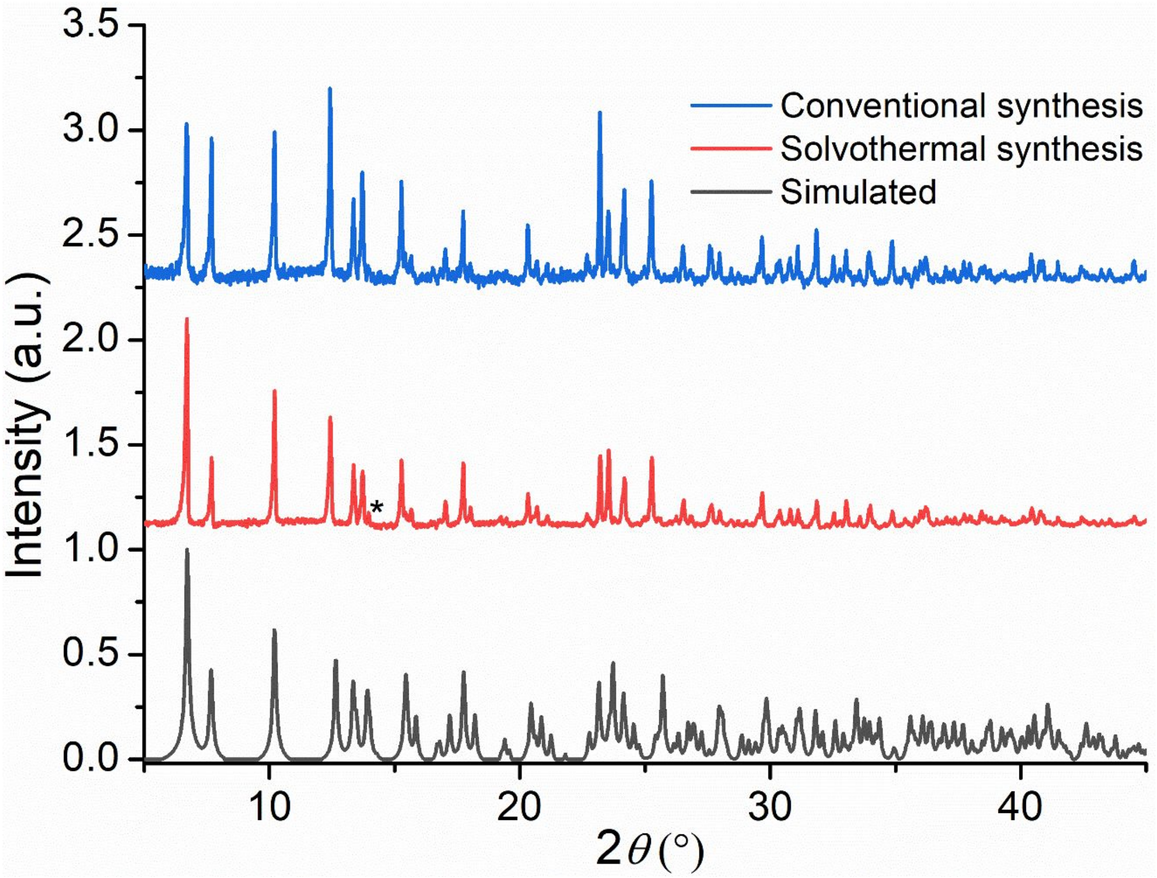

Figure S22. The comparison of experimentally collected and simulated PXRD patterns of complex **2**. The marked diffractions peaks (*) correspond to impurities in form of reagents and unidentified byproducts in **2**.

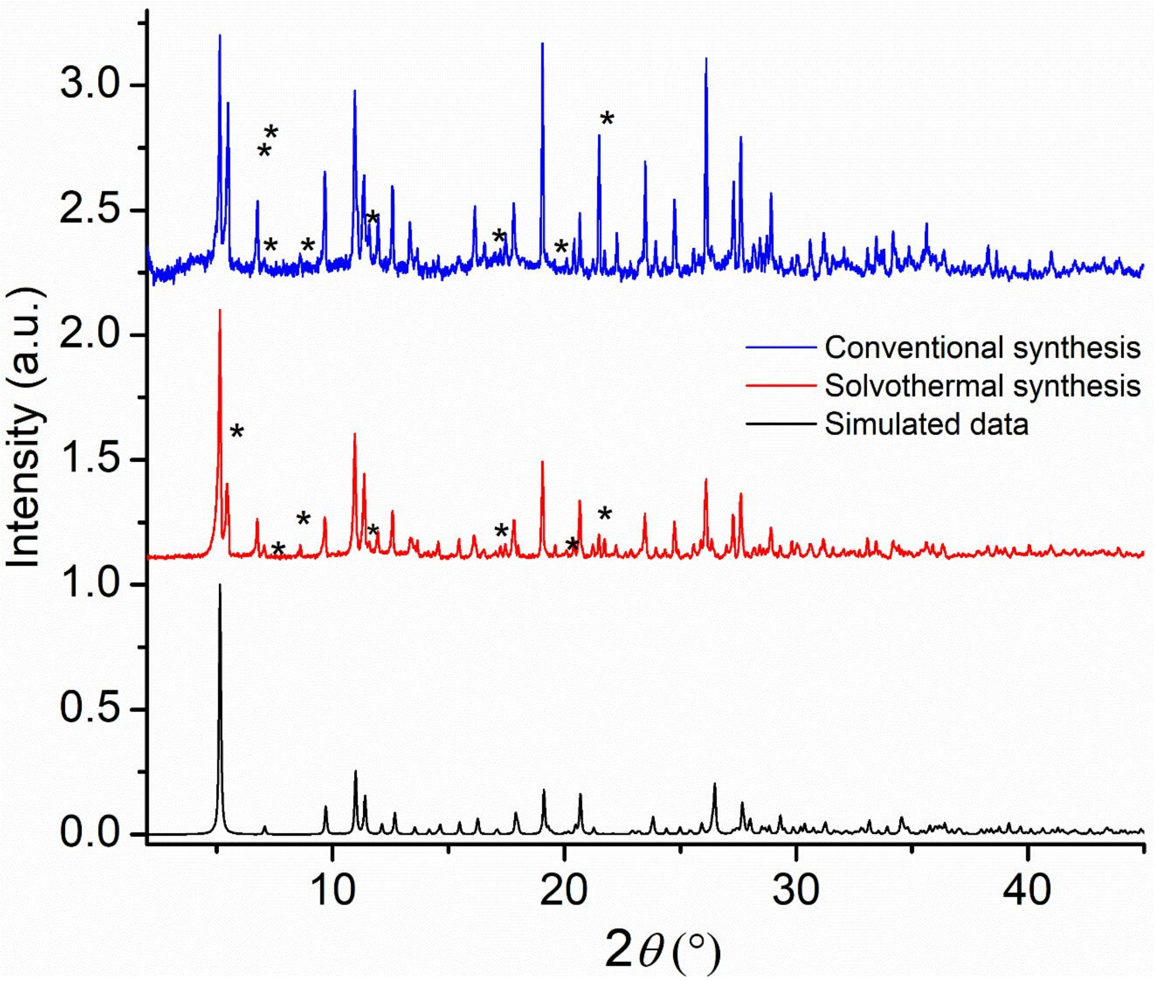

Figure S23. The comparison of experimentally collected and simulated PXRD patterns of complex **3**. The marked diffractions peaks (*) correspond to impurities in form of reagents and unidentified byproducts or alternative crystal forms of **3**.

5. Spectroscopic data

Table S3. Photophysical properties of the complexes **1**, **2**, and **3** in THF.

	λ_{abs}, nm	λ_{em}, nm	τ_{Fl}, ns	Φ %
1	391<br>336	462	0.21 (15.7%), 0.45 (84.3%)	3.27
2	392<br>335	462	0.24 (20.1%), 0.46 (79.9%)	2.36
3	392<br>335	462	0.25 (22.7%), 0.47 (77.3%)	1.95

Table S4. Photophysical properties of the complexes **1**, **2**, and **3** in ACN.

	λ_{abs}, nm	λ_{em}, nm	τ_{Fl}, ns	Φ %
**1**	389<br>335	468	0.11 (27.5%), 0.42 (72.5%)	1.62
**2**	388<br>335	467	0.12 (13.1%), 0.42 (86.9%)	0.98
**3**	456<br>382<br>332	469	0.2 (14.9%), 0.43 (85.1%)	0.56

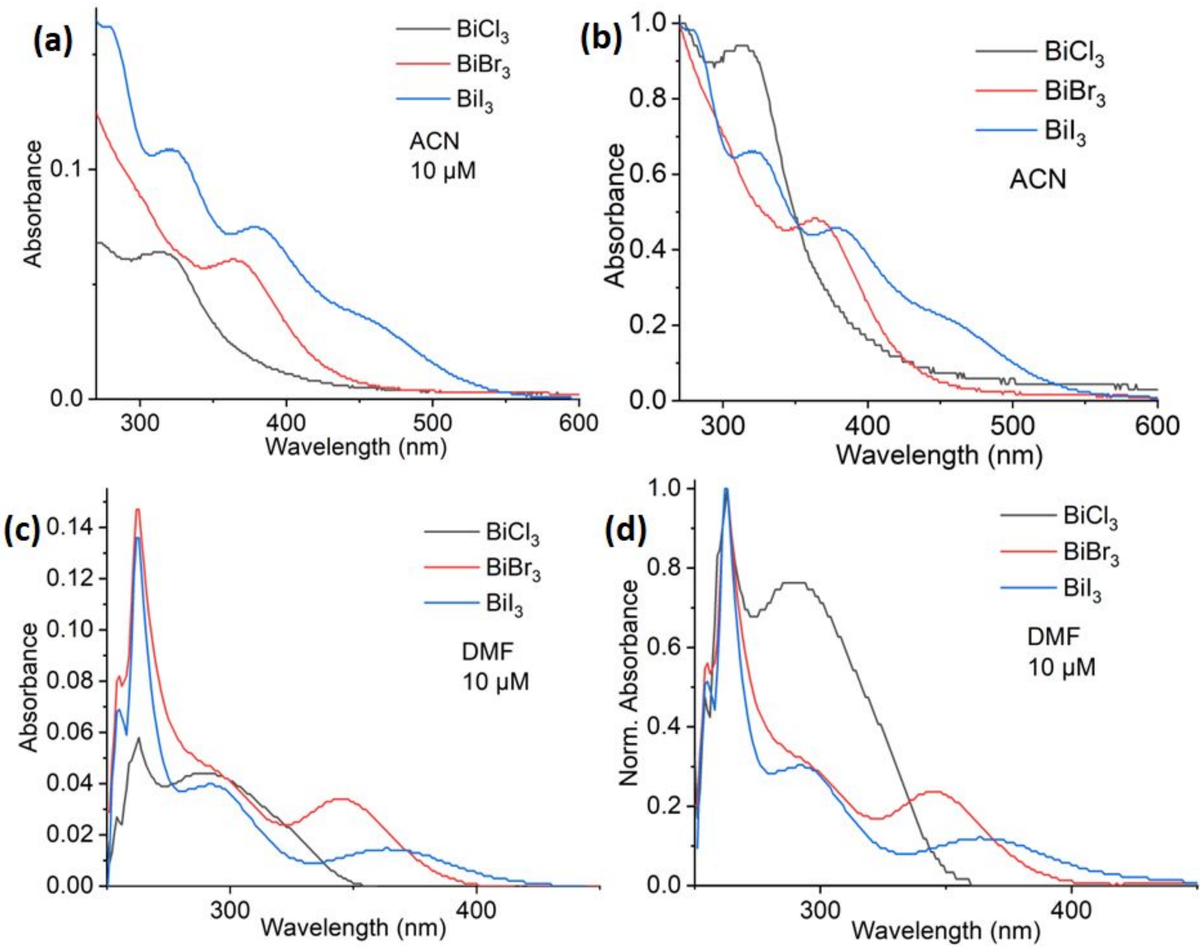

Figure S24. The comparison of the absortion spectra of $BiCl_3$, $BiBr_3$, and BiI_3 in (a,b) ACN and (c,d) DMF at 10 μM under ambient conditions.

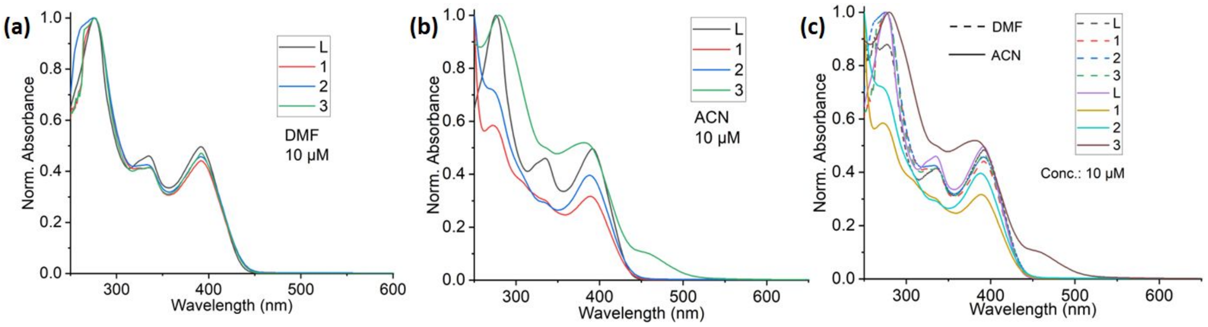

Figure S25. The normalized absorption spectra of complexes, and ligand recorded in DMF (a) and ACN (b) at 10 μM under ambient conditions. (c) Comparison of normalized absorption spectra recorded in DMF and ACN.

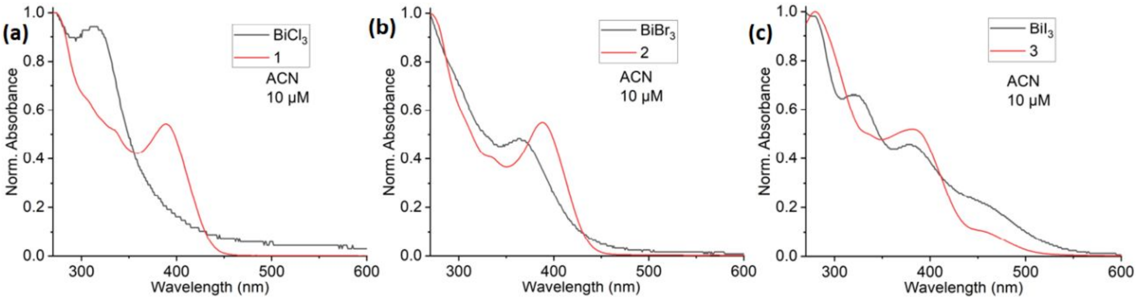

Figure S26. The comparison of individual absorption spectra of bismuth salts and complexes (a) **1**, (b) **2** and (c) **3** recorded in ACN at concentration of 10 μM under ambient conditions.

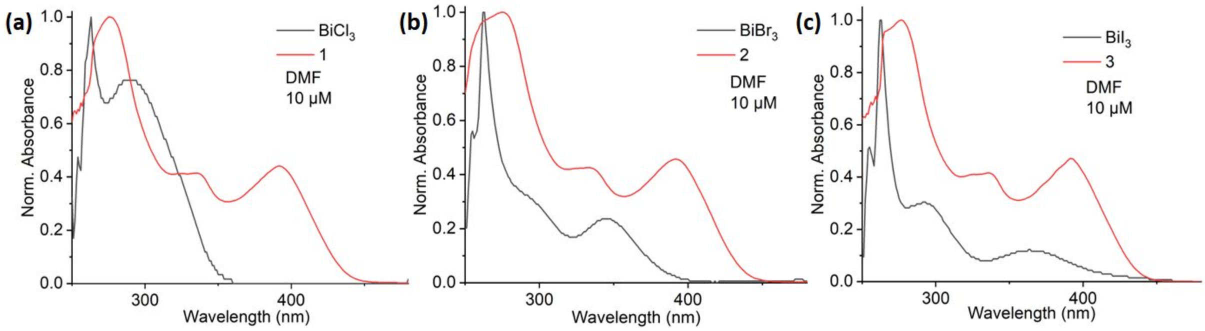

Figure S27. The comparison of individual absorption spectra of bismuth salts and complexes (a) **1**, (b) **2** and (c) **3** recorded in DMF at concentration of 10 μM under ambient conditions.

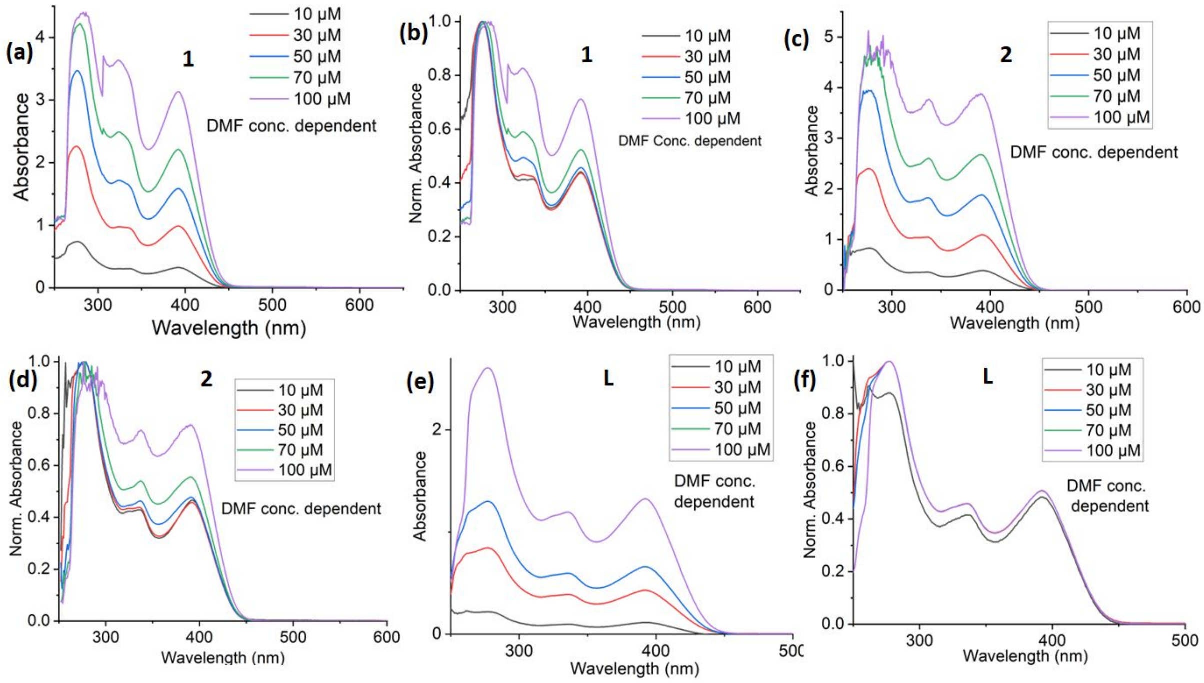

Figure S28. The concentration dependent absorption spectra of **1** (a, b), **2** (c, d) and **L** (e, f) recorded in DMF at concentration of 10 µM under ambient conditions.

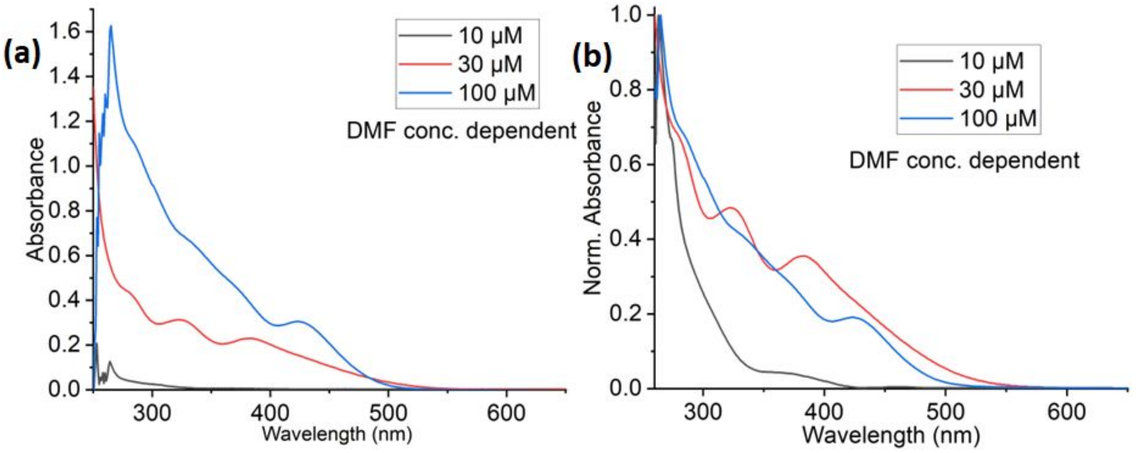

Figure S29. The concentration dependent (a) and normalized (b) absorption spectra of BiI_3 recorded in DMF under ambient conditions.

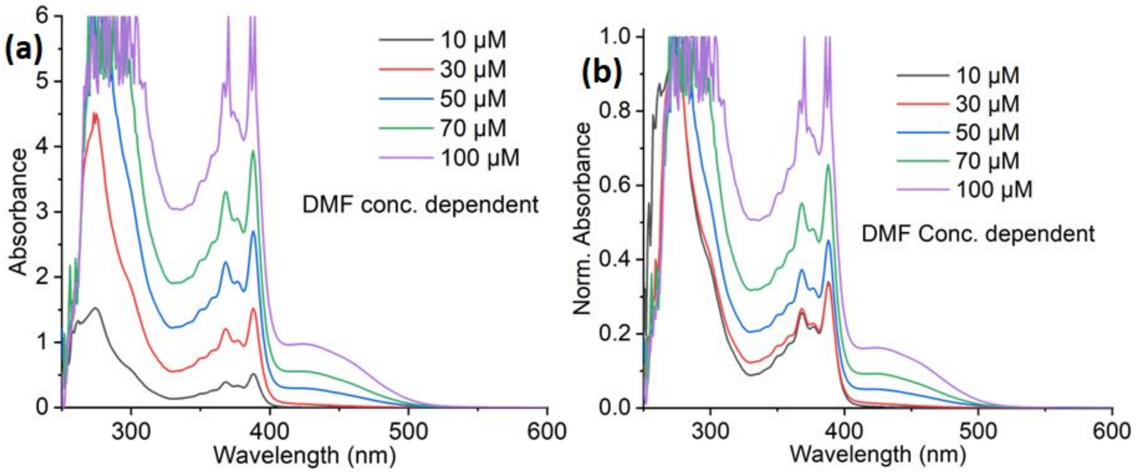

Figure S30. The concentration dependent (a) and normalized (b) absorption spectra of **4** recorded in under ambient conditions.

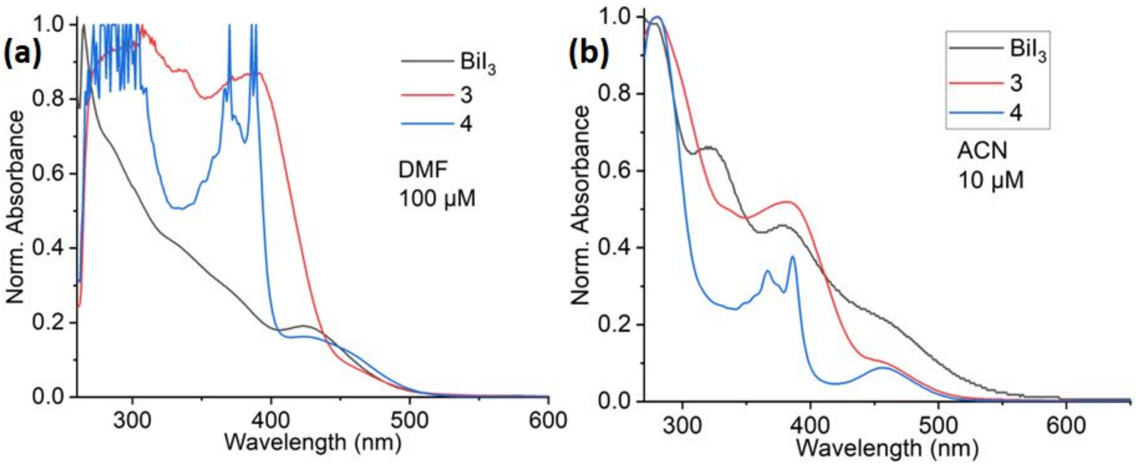

Figure S31. The comparison of normalized absorption spectra of BiI_3, **3** and **4** recorded under ambient conditions in DMF (a) and ACN (b).

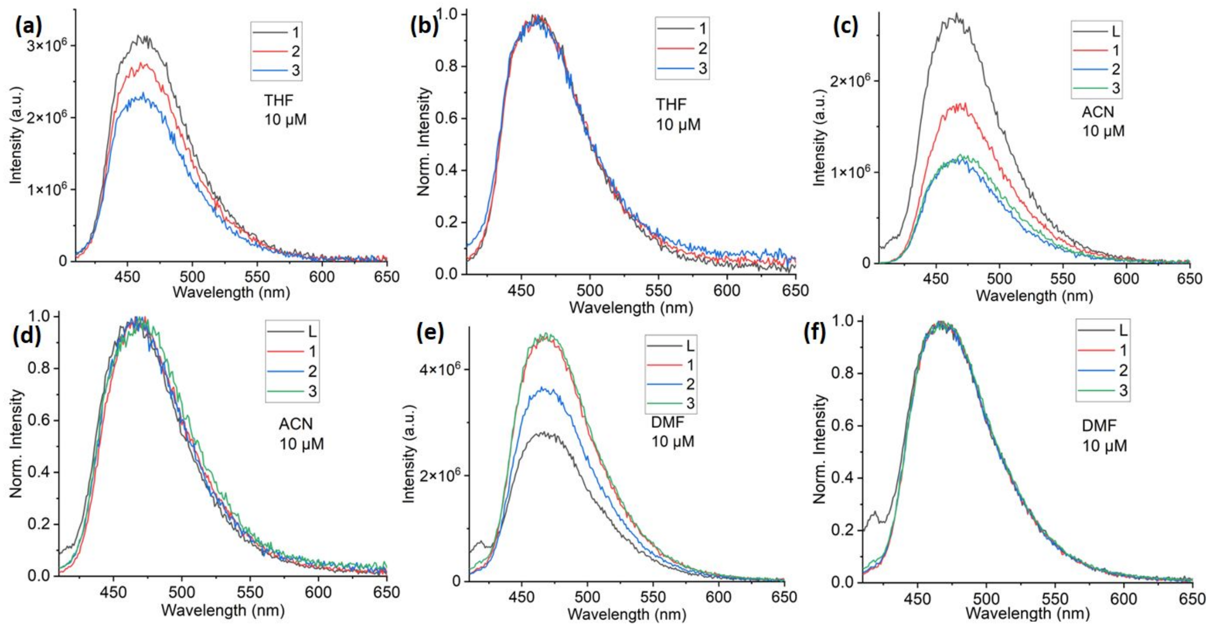

Figure S32. The comparison of the photoluminescence emission spectra of **1**, **2**, **3** and **L** in solvents of different polarity, THF (a, b), ACN (c, d) and DMF (e, f), at the excitation of 392 nm under ambient conditions (conc. = 10 μM).

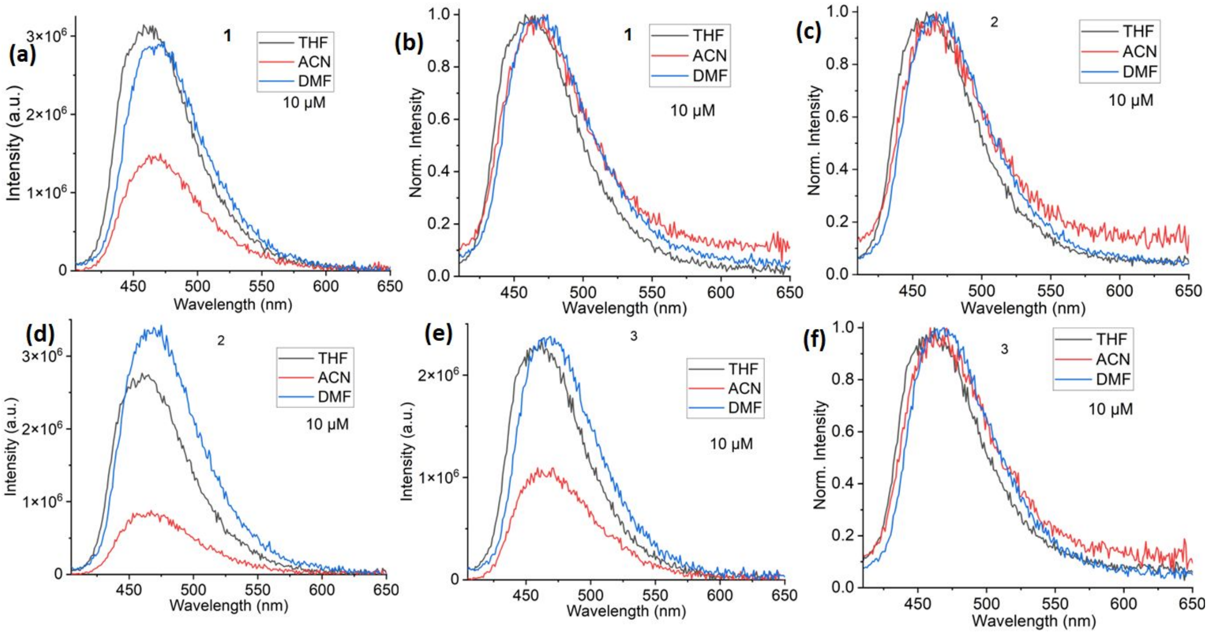

Figure S33. The comparison of the photolumnescence emission spectra of (a ,b) **1**, (c, d) **2**, (e, f) **3** in different solvents at the excitation of 392 nm under ambient conditions (conc. = 10 μM).

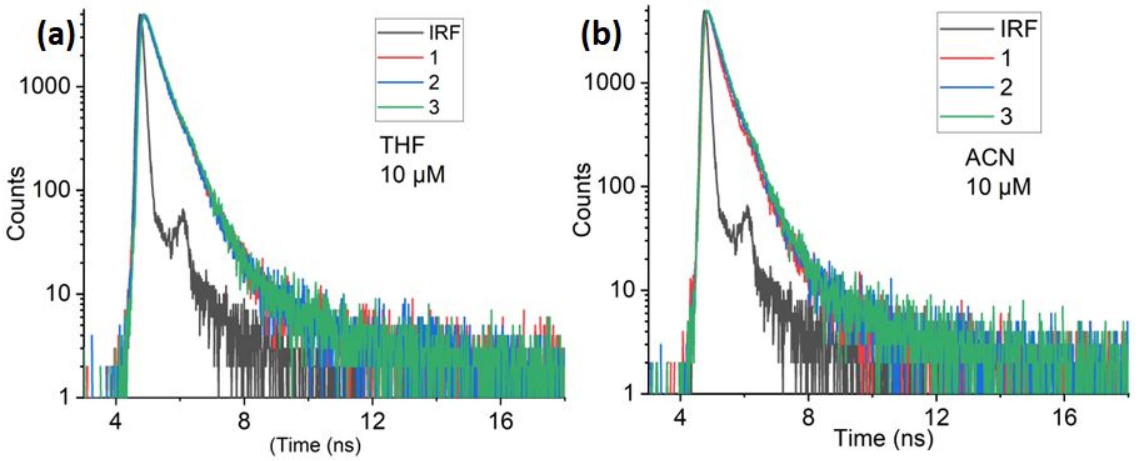

Figure S34. The fluorescence lifetime of the comlexes recored in (a) THF, and (b) ACN under ambient conditions at the excitation of 375.6 nm (conc. = 10 μM).

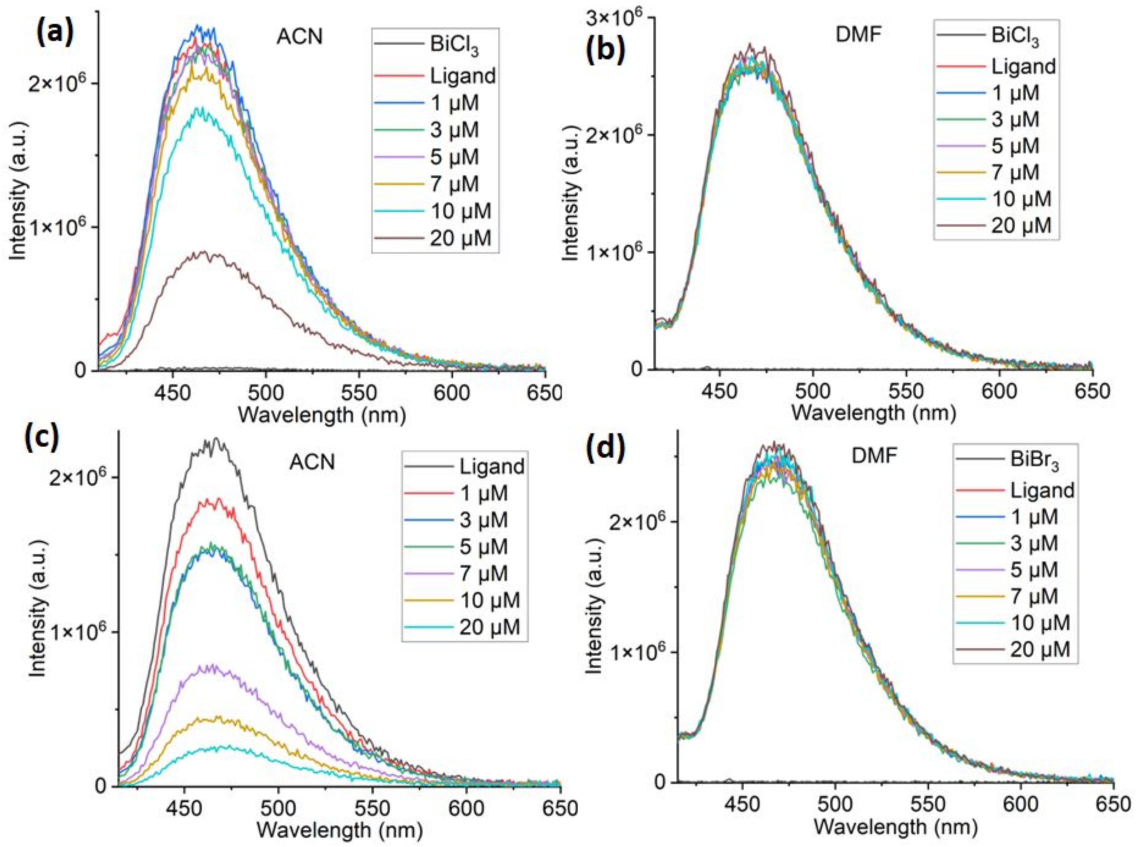

Figure S35. The titration study of **L** with increasing the amount of $BiCl_3$ and $BiBr_3$ in (a,c) ACN, and (b, d) DMF respectively at excitation of 392 nm under ambient conditions.

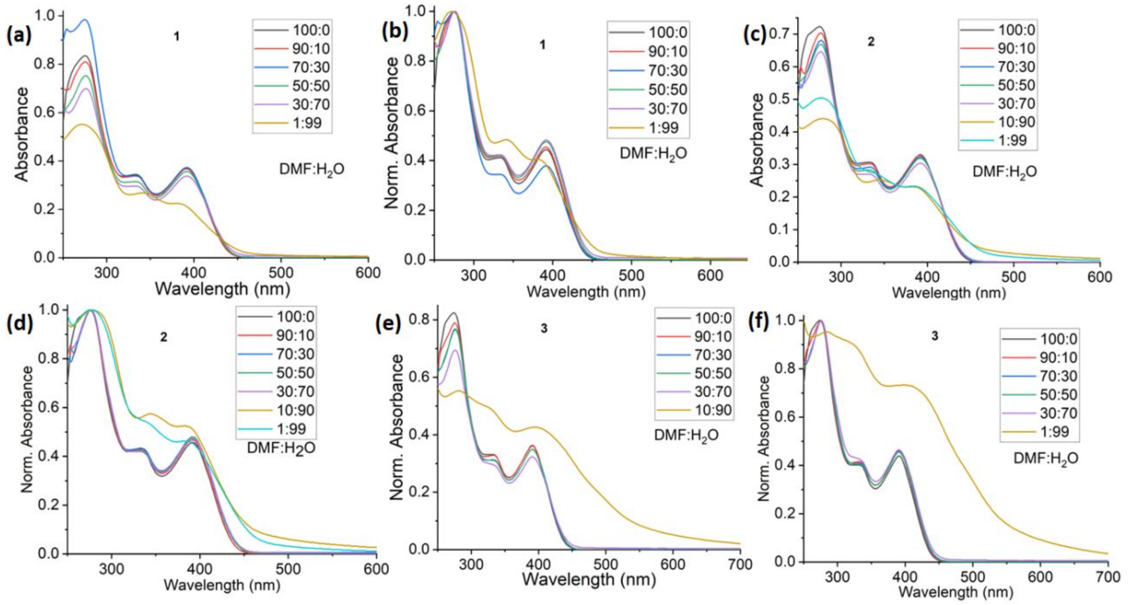

Figure S36. The comaprison of the absorption spectra of complexes (a, b) **1**, (c, d) **2** and (e, f) **3** with the increase in the amount of water at concentration of 10 μM under ambient conditions.

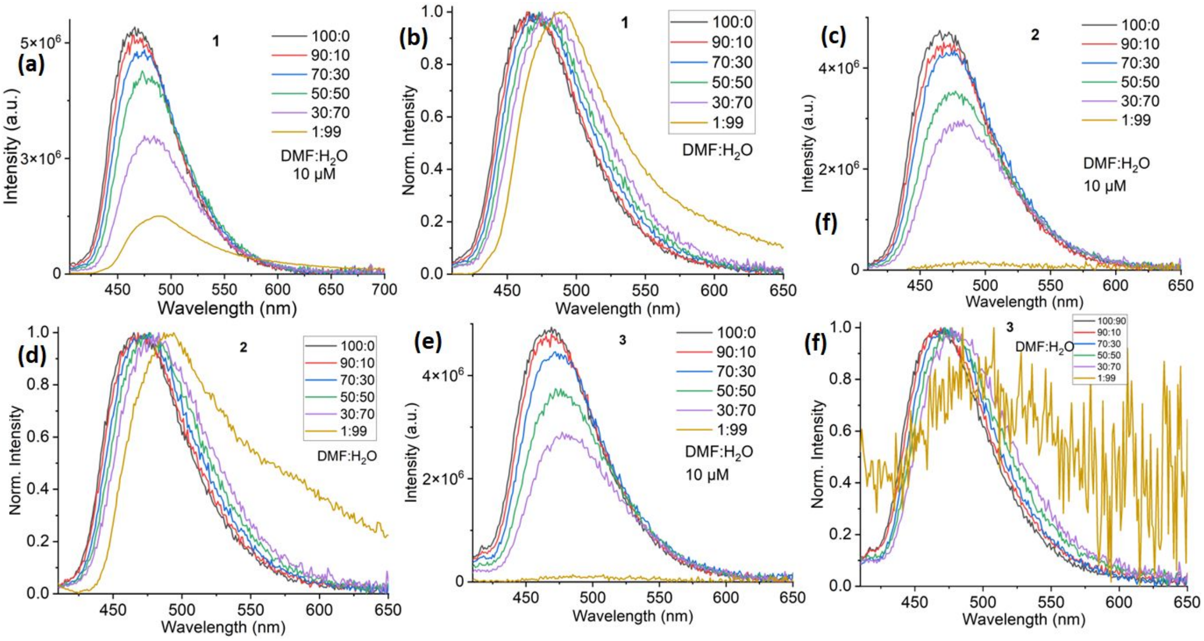

Figure S37. The comaprison of the photoluminescence spectra of the complexes (a, b) **1**, (c, d) **2** and (e, f) **3** with the increase in the amount of water at the excitation of 375.4 nm (conc. = 10 μM) under ambient conditions.

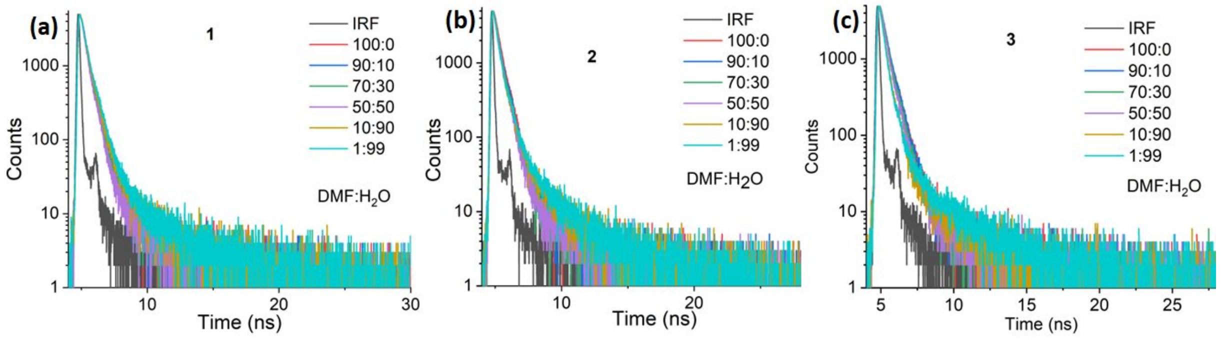

Figure S38. The comaprison of the fluorescence lifetimes of complexes (a) **1**, (b) **2** and (c) **3** with the increase in the amount of water to DMF at the excitation of 375.4 nm (conc. = 10 μM) under ambient conditions.

Table S5. Table for fluorescence lifetimes of aggregates for complexes **1**, **2**, and **3** in DMF and water mixture.

**DMF:H_2O**	**τ(1) (ns)**	**τ(2) (ns)**	**τ(3) (ns)**
**100:0**	0.32(23.1%), 0.47(76.9%)	0.38(67.6%), 0.53(32.4%)	0.36(49.8%), 0.50(50.2%)
**90:10**	0.28(15.6%), 0.44(84.4%)	0.33(41.9%), 0.48(58.1%)	0.38(71.8%), 0.52(28.2%)
**70:30**	0.28(18.5%), 0.41(81.5)	0.33(56.8%), 0.47(43.2%)	0.36(76.4%), 0.52(23.6%)
**50:50**	0.23(10.5%), 0.38(89.5%)	0.34(90.1%), 0.54(9.9%)	0.35(95.8%), 0.66(4.2%)
**10:90**	0.40(94.9%), 1.47(5.1%)	0.33(91.8%), 1.51(8.2%)	0.3(97.9%), 2.08(2.1%)
**1:99**	0.42(92.7%), 1.32(7.3%)	0.33(89%), 1.42(11%)	0.28(95.7%), 2.16(4.3%)

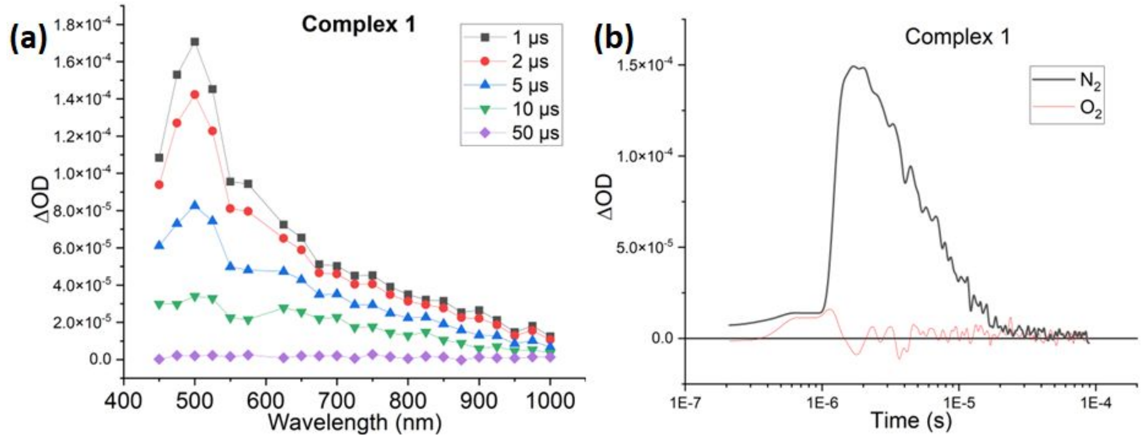

Figure S39. (a) The μs-TA spectra for complex **1** solution under nitrogen atomsphere with probe delay time from 1-50 μs and excitation density 11 μJ cm^{-2}.(b) oxygen quenching TA decay for complex **1** solution probing at 500 nm, under nitrogen atomsphere and followed with oxygen.

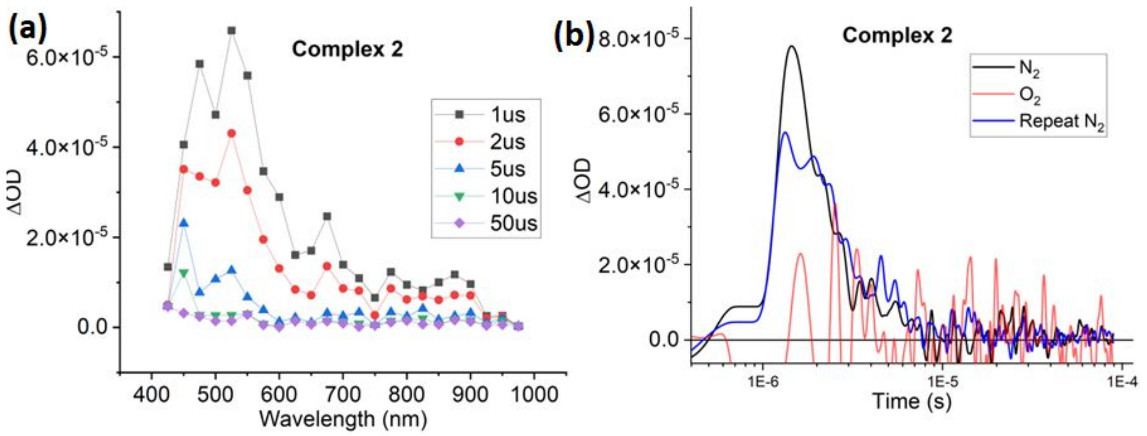

Figure S40 (a) μs-TA spectra for complex 2 solution under nitrogen atomsphere with prob delay time from 1 - 50 μs and excitation density 11 μJ cm-2. (b)oxygen quenchingTA decay for complex 2 solution probing at 500 nm, under the order of notrogen, follwed with oxygen and repeat recovery in nitrogen atomsphere.

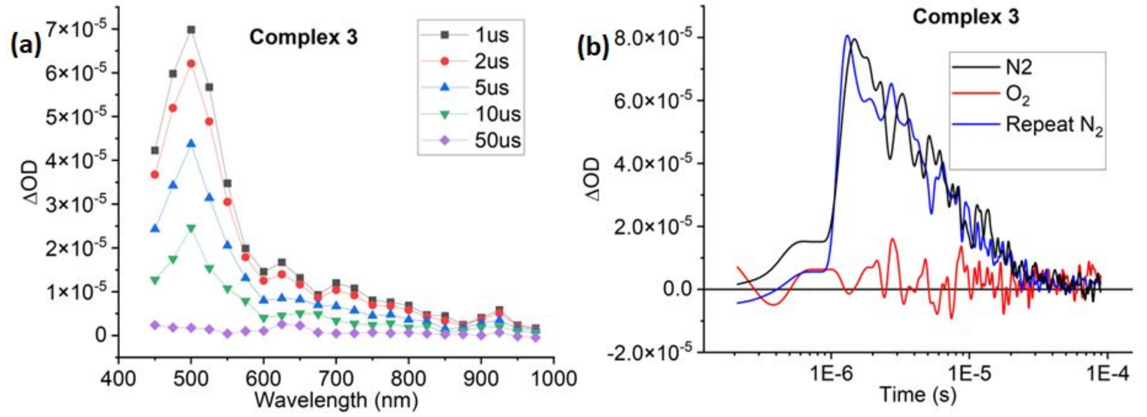

Figure S41. (a) μs-TA spectra for complex 3 solution under nitrogen atomsphere with prob delay time from 1 - 50 μs and excitation density 11 μJ cm-2. (b)oxygen quenchingTA decay for complex 3 solution probing at 500 nm, under the order of notrogen, follwed with oxygen and repeat recovery in nitrogen atomsphere.

6. Density functional theory

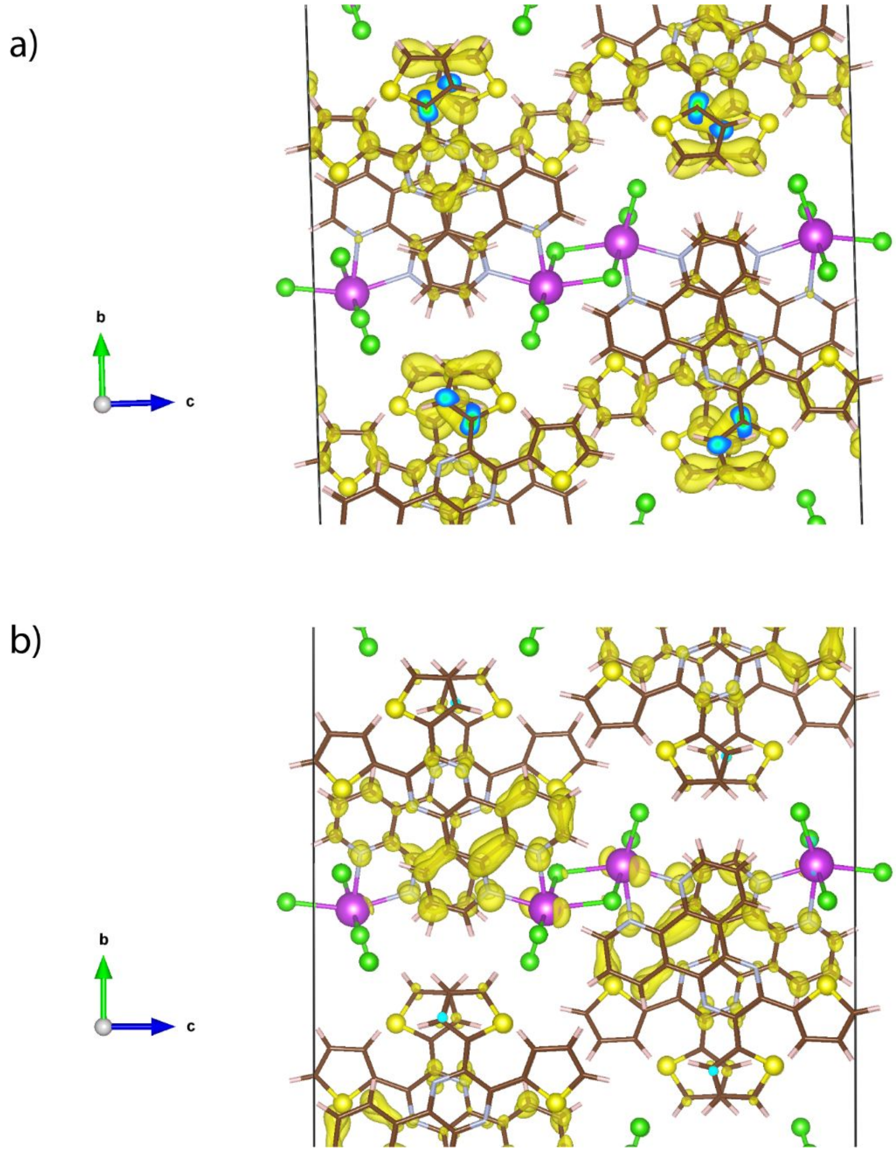

Figure S42. Partial charge density isosurfaces (yellow) of complex 1 at 8 × 10^{-5} e/Å^3: (a) valence band maximum (b) conduction band minimum. Bi atoms are in purple, Cl in green, C in brown, H in pink, N in blue – organic ligand atoms are depicted in line style for clarity. Plotted using the VESTA software package.[6]

7. Ultraviolet photoelectron spectroscopy

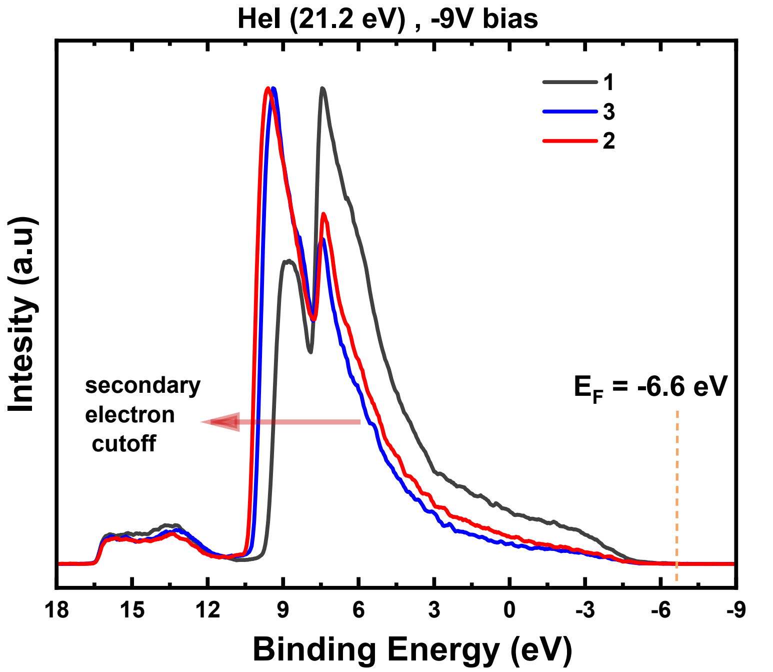

Figure S43. Photoelectron spectra of complexes **1**, **2** and **3** plotted as a function of binding energy.

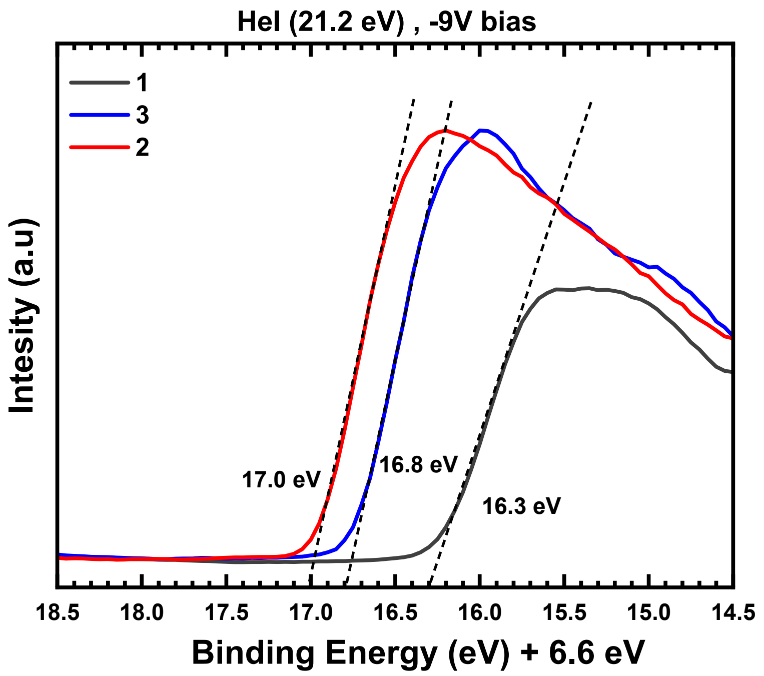

Figure S44. Expansion of the region near the vacuum level cutoff with respect to the Fermi level. The tangent lines determinining the value of the SECO.

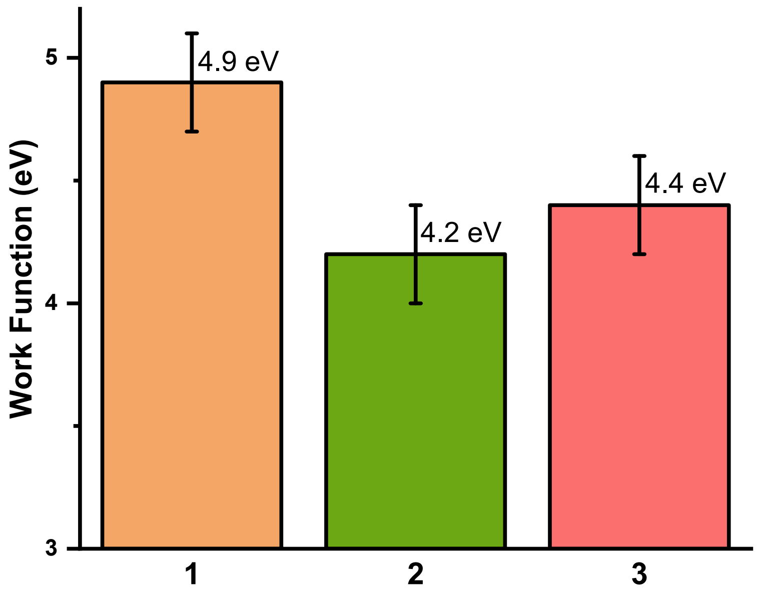

Figure S45. Calculated workfucntions for complexes **1**, **2** and **3**, repsectively, extracted from the UPS data.

8. References

1. *CrysAllisPro*, *Agilent Technologies Inc.*, 2022.
2. G. M. Sheldrick, *SHELXT* - Integrated space-group and crystal-structure determination. *Acta Crystallogr A.*, 2015, **64***,* 3–8.
3. O. V. Dolomanov, L. J. Bourhis, R. J. Gildea, J. A. K. Howard, H. Puschmann, *OLEX2*: a complete structure solution, refinement and analysis program., *J. Appl. Crystallogr.* 2009, **42**, 339–341.
4. G. M Sheldrick, Crystal structure refinement with *SHELXL*. *Acta Crystallogr C.*, 2015, **71***,* 3–8.
5. C. B. Hübschle, G. M. Sheldrick and B. Dittrich, ShelXle: a Qt graphical user interface for SHELXL, *J. Appl. Cryst.*, 2011, **44**, 1281–1284.
